# Identification and Characterization of Genes Related to the Prognosis of Hepatocellular Carcinoma Based on Single-Cell Sequencing

**DOI:** 10.3389/pore.2022.1610199

**Published:** 2022-08-25

**Authors:** Wenbiao Chen, Feng Zhang, Huixuan Xu, Xianliang Hou, Donge Tang, Yong Dai

**Affiliations:** ^1^ Research Center for Human Tissue and Organs Degeneration, Institute of Biomedicine and Biotechnology, Shenzhen Institutes of Advanced Technology, Chinese Academy of Sciences, Shenzhen, China; ^2^ Department of Respiratory Medicine, People’s Hospital of Longhua, The Affiliated Hospital of Southern Medical University, Shenzhen, China; ^3^ Clinical Medical Research Center, Guangdong Provincial Engineering Research Center of Autoimmune Disease Precision Medicine, The Second Clinical Medical College of Jinan University, Shenzhen People’s Hospital, Shenzhen, China; ^4^ Central Laboratory, People’s Hospital of Longhua, The Affiliated Hospital of Southern Medical University, Shenzhen, China; ^5^ Intensive Care Unit, The First Affiliated Hospital of Jinan University, Guangzhou, China

**Keywords:** gene, hepatocellular carcinoma, prognostic model, single-cell sequencing, molecular cluster

## Abstract

The heterogeneity of hepatocellular carcinoma (HCC) highlights the importance of precision therapy. In recent years, single-cell RNA sequencing has been used to reveal the expression of genes at the single-cell level and comprehensively study cell heterogeneity. This study combined big data analytics and single-cell data mining to study the influence of genes on HCC prognosis. The cells and genes closely related to the HCC were screened through single-cell RNA sequencing (71,915 cells, including 34,414 tumor cells) and big data analysis. Comprehensive bioinformatics analysis of the key genes of HCC was conducted for molecular classification and multi-dimensional correlation analyses, and a prognostic model for HCC was established. Finally, the correlation between the prognostic model and clinicopathological features was analyzed. 16,880 specific cells, screened from the single-cell expression profile matrix, were divided into 20 sub-clusters. Cell typing revealed that 97% of these cells corresponded to HCC cell lines, demonstrating the high specificity of cells derived from single-cell sequencing. 2,038 genes with high variability were obtained. The 371 HCC samples were divided into two molecular clusters. Cluster 1 (C1) was associated with tumorigenesis, high immune score, immunotherapy targets (PD-L1 and CYLA-4), high pathological stage, and poor prognosis. Cluster 2 (C2) was related to metabolic and immune function, low immune score, low pathological stage, and good prognosis. Seven differentially expressed genes (CYP3A4, NR1I2, CYP2C9, TTR, APOC3, CYP1A2, and AFP) identified between the two molecular clusters were used to construct a prognostic model. We further validated the correlation between the seven key genes and clinical features, and the established prognostic model could effectively predict HCC prognosis. Our study identified seven key genes related to HCC that were used to construct a prognostic model through single-cell sequencing and big data analytics. This study provides new insights for further research on clinical targets of HCC and new biomarkers for clinical application.

## Introduction

Over the years, hepatocellular carcinoma (HCC) has become a major public health concern, given that it is one of the most common malignant tumors globally, ranking sixth in incidence and third in mortality [1]. The treatment of HCC encompasses traditional surgery, radiotherapy, chemotherapy, immunotherapy and targeted therapy [2]. Despite unprecedented advances in diagnostic and therapeutic approaches in recent years, the overall mortality rate of HCC remains dismal. Most importantly, patients are often diagnosed at advanced or terminal stages and are not indicated for effective treatment methods such as surgery, accounting for the poor prognosis [2, 3]. The early symptoms of HCC are largely unspecific, and there is currently a lack of clinical markers to help clinicians for early diagnosis and treatment [2], emphasizing the need to discover novel molecular targets to improve the current clinical management of HCC.

HCC heterogeneity can determine patient outcomes; indeed, HCC tumor cells and tissues from different patients may exhibit different characteristics that account for the difference in sensitivity to treatment [4]. During tumor proliferation and division, the primary cells undergo a change in molecular and genetic components, resulting in altered molecular characteristics of the tumor cell. These changes account for the differences in treatment response and prognosis of tumors [5], explaining the heterogeneity in response to immunotherapy and targeted therapy on HCC during clinical practice [6]. High-throughput sequencing technology has been widely used in various fields of biology and medicine and has substantially contributed to enhancing the current understanding of tumor heterogeneity [7]. However, traditional bulk RNA-sequence is based on tissue samples (cell populations), which reflect the average gene expression level in the cell population, and could have huge significance for the design of targeted therapy [7, 8]. In recent years, significant inroads have been achieved in single-cell RNA sequencing technology to reveal the expression of all genes in the whole genome at the single-cell level and obtain more robust and objective results on tumor cell heterogeneity [9]. Bioinformatics technology can play a complementary role to single-cell sequencing. Bioinformatics analysis based on single-cell sequencing data can enable us to explore the structure and function of genes more accurately and enhance the molecular prediction efficacy of gene targets for new cancer drugs [8]. In silico analyses also enable correlation analysis between predicted targets and clinical features of tumors and validate the efficacy of gene targets for tumor diagnosis, treatment, and prognosis assessment [10].

In this study, to avoid the impact of HCC heterogeneity on target mining, we integrated single-cell sequencing and bioinformatics analysis using publicly available datasets to identify gene targets related to HCC clinical features. The HCC samples could be divided into 2 clusters based on single-cell sequencing genes. Both clusters exhibited distinct characteristics in terms of clinical features, immune function, and molecular pathways. Besides, seven genes closely associated with the clinical features were identified and were used to construct the prognostic model, which exhibited good predictive efficacy in HCC patients (Supplementary Figure S1). Our work based on single-cell sequencing and bioinformatics analysis of big data will deepen our understanding of the heterogeneity in HCC cell characteristics and may provide effective targets to improve clinical diagnosis and treatment of HCC.

## Methods

### Data Acquisition and Preprocessing

The normalized single-cell sequencing data on 10 HCC samples and 71,915 cells were acquired from the Gene Expression Omnibus (GEO) dataset GSE149614. The RNA-sequence and clinical information of 371 HCC patients were downloaded from The Cancer Genome Atlas (TCGA) database. The GSE14520 dataset of HCC samples (*n* = 242) was used for validation. Then, the RNA sequence was transformed from fragments per kilobase of million reads (FPKM) to Transcripts Per Kilobase Million (TPM).

### Single-Cell Sequencing Recognition and Analysis

34,414 cancer cells were screened from the single-cell expression profile matrix of 71,915 cells using the Seurat R package (version 3.0.1). The cancer cells were selected in this study after eliminating cells with low and high expression (each gene was expressed in at least 100 cells, and at least 2,000 genes were detected expression in each cell). Then, the single-cell sequencing data was standardized using the NormalizeData algorithm, and the FindVariableFeatures function was used to screen for high variation genes. Next, the high variation genes were conducted to Z-score algorithm for standardization analysis. The RunPCA algorithm was used to reduce the dimensionality of the dataset, and the top 10 principal components (PC) were used for further nonlinear dimension reduction using the UMAP algorithm. Subsequently, the main clusters were identified using the FindNeighbors and FindClusters algorithms. The key genes were selected after dimensionality reduction. Then the FindAllMarkers function was used to screen marker genes of each cell cluster. Next, the R package monocle was used for cell trajectory analysis, and the cluster map was generated based on the single-cell expression profile of marker genes. The DDRTree method was used to reduce the dimensionality of these key genes, and cell trajectories were plotted against cell clusters. The online database Human Cell Landscape (http://bis.zju.edu.cn/HCL/) was used to analyze important genes in tumor cells from single-cell sequencing data and select cell types with the highest cell correlation for further analysis.

### Molecular Classification Identification

We extracted the expression profile matrix of key genes identified by single-cell sequencing from TCGA database, consisting of 371 HCC samples and 1,930 genes. Next, a non-negative matrix clustering algorithm (NMF) was used to conduct unsupervised clustering on 371 HCC samples corresponding to 1,930 genes. The number of clusters k was set to 2–10, the average contour width of the common member matrix was determined through the R package NMF, and the minimum number of members in each subclass was set to 10. The optimal k value was determined by cophenetic, dispersion and silhouette metrics. The different survival outcomes associated with our molecular classification were evaluated by Kaplan-Meier (KM) analysis and the log-rank test using R package survival. Gene set variation analysis (GSVA) was performed to determine pathway activity scores of each molecular group using the R package GSVA.

### Immune in Infiltration and Checkpoint Analyses

To study the composition of immune cells in the molecular groups, the CIBERSORT (http://cibersort.stanford.edu/) tool was used to calculate the immune scores of 22 immune cells for molecular classification using the LM22 signature as a reference. Furthermore, we used the ESTIMATE (https://sourceforge.net/projects/estimateproject/) algorithm to evaluate the stromal and immune scores of malignant tumor tissue based on expression data. Stromal and immune fractions were analyzed to predict the levels of stromal and immune cells in tumor tissue, respectively. StromalScore, ImmuneScore, and ESTIMATEScore were used to calculate each sample score. These scores were then compared across molecular groups and verified by Kruskal-Wallis Test. Moreover, we analyzed the distribution of immunotherapy targets of PD-L1 programmed cell death protein 1 (PD-L1) and cytotoxic T lymphocyte-associated antigen-4 (CTLA-4) across molecular groups.

### Gene Set Enrichment Analysis on Molecular Classification

GSEA analysis was performed to detect which gene sets were significantly enriched in each molecular classification using the R package clusterProfiler. We selected the c2.cp.kegg.v7.0.symbols.gmt gene set as a reference, which contained the KEGG pathway within. The GSEA input file contained expression profile data and molecular cluster tags. Sample labels were used to mark the samples belonging to their respective molecular groups. The enriched signaling pathways with a false discovery rate (FDR) < 0.05 were statistically significant.

### Screening of Key Genes in Molecular Classification and Construction of Prognostic Model

The differentially expressed genes (DEGs) between molecular groups were analyzed by R package Limma. The fold change between molecular groups was calculated using an empirical Bayesian method and identified by the consensus clustering method using moderated t-tests. The Benjamini–Hochberg correction adjusted the *p*-value for multiple testing. DEGs with a false discovery rate (FDR) < 0.05 and fold change >2 were identified between the molecular groups. We identified interactions among key genes using the online database STRING (https://string-db.org/) and set the minimum required Interaction score to 400. The protein-protein interaction networks of important genes were generated using Cytoscape. Moreover, the R function “cor.test” was used to calculate and test the correlation coefficient. Next, UniCox and LASSO-Cox algorithms were applied to reduce dimensionality, and the Cox Proportional-Hazards prognostic model was established using the screened key genes.

### Validation of Keys Genes and Prognostic Model

We extracted the expression matrix and clinical prognosis information of key genes and divided the HCC samples into high and low expression groups according to the median expression value of each gene. KM method was used for survival analysis, and the log-rank test was used for comparison. The sensitivity and specificity of the prognostic model were evaluated by receiver operating characteristic (ROC) curves. Next, we used these key genes as a signature to predict the overall survival (OS) time of patients. We calculated the risk score of each sample according to the expression level of key genes in each sample and plotted the risk score distribution. The HCC samples were divided into high-risk and low-risk groups based on the median risk score, the KM method was used for survival analysis, and the log-rank test was used to compare survival times. The association between the prognostic model and clinical features was analyzed by univariate and multivariate analysis. Using the R package RMS, we constructed nomograms to compare the risk-score between the prognostic model and clinical features. Calibration curve analysis was conducted to evaluate the performance of the prognostic model. Moreover, univariate and multivariate logistic analyses using Cox proportional hazards regression were performed to assess the relationship between the risk score of the prognostic model and clinical features. We analyzed the differences in response to immunotherapy and chemotherapy among molecular clusters. Subclass mapping was used to compare the similarity between the molecular clusters and immunotherapy patients in the IMvigor210 dataset (The lower the *p*-value, the higher the similarity). The Hazard ratios (HR) and 95% confidence intervals (CI) of the prognostic model and other clinical features were also calculated.

### Statistical Analysis

Chi-square analysis was used to evaluate the relationship between molecular classification and clinical features. The unpaired Student’s t-test and the Mann-Whitney U-test were used for comparing two groups with normally distributed and non-parametric variables. When comparing three groups, one-way analysis of variance and Kruskal–Wallis tests were used for parametric and non-parametric data. The Concordance index (C-index) was used to assess the accuracy of the prognostic model by multivariate Cox regression analysis. All analyses in this study were performed using R software (version 3.5.1) and SPSS software (version 24). A two-sided *p*-value <0.05 was statistically significant. The R code used for the data analysis were submitted as Supplementary Table S1.

## Results

### Screening of HCC Related-Cells and Genes Based on Single-Cell RNA Sequencing

**FIGURE 1:**
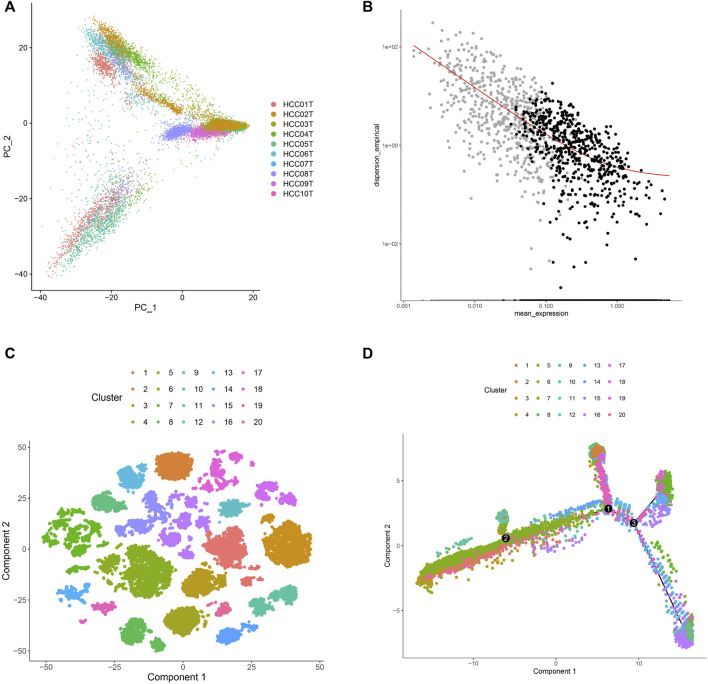
Characterization of single-cell RNA sequencing and screening of marker genes. **(A)** UMAP clustering was used to classify the cell groups into sub-clusters. **(B)** The screened marker genes of cells. The black dots were the screened marker genes. **(C)** The single-cell expression profile of 2,038 marker genes obtained by dimensionality reduction was used to map the distribution of 20 cell sub-clusters. **(D)** Trajectory analysis colored of 20 cell sub-clusters.

In this study, 71,915 cells from 10 HCC samples were analyzed by single-cell RNA sequencing. Of these, 34,414 cells were selected based on the single-cell expression matrix. Subsequently, 16,880 cells were screened after quality control which involved the removal of cells with low and high expression (Supplementary Figure S2), which yielded 15,093 genes with high variable expression (Supplementary Figure S3). The 15,093 cells were divided into 20 sub-clusters after dimensionality reduction and cluster analyses (Figure 1A), which suggested high single-cell heterogeneity of HCC cells in this study. In addition to the 15,093 cells with highly variable expression, we identified 4,121 marker genes. After removing duplicate genes, 2,038 marker genes were left for cell sub-clustering, cell annotation, and trajectory analysis (Figure 1B). The 15,093 cells were divided into 20 sub-clusters consisting of primary and metastatic cells, revealing high clustering of cells from each sample (Figure 1C). Moreover, we annotated the sub-clusters for each cell sample using the marker genes and plotted the trajectory of the 20 sub-clusters. The results showed that the underlying transcriptional heterogeneity across sub-clusters and the cells of sub-clusters were scattered (Figure 1D). In addition, to characterize the 20 sub-clusters, we analyzed the 2,038 genes and 15,093 cells and selected cell types with the highest correlations. The results revealed that 15,093 cells could be divided into 72 cell types. Furthermore, we investigated the cell type proportions in the 20 sub-clusters and found that 97% of them were HCC cells (Supplementary Table S2). These results revealed that HCC cells screened by single-cell RNA sequencing had good homology, conducive to obtaining a more accurate molecular classification.

### Molecular Classification Based on Single-Cell RNA Sequencing

**FIGURE 2:**
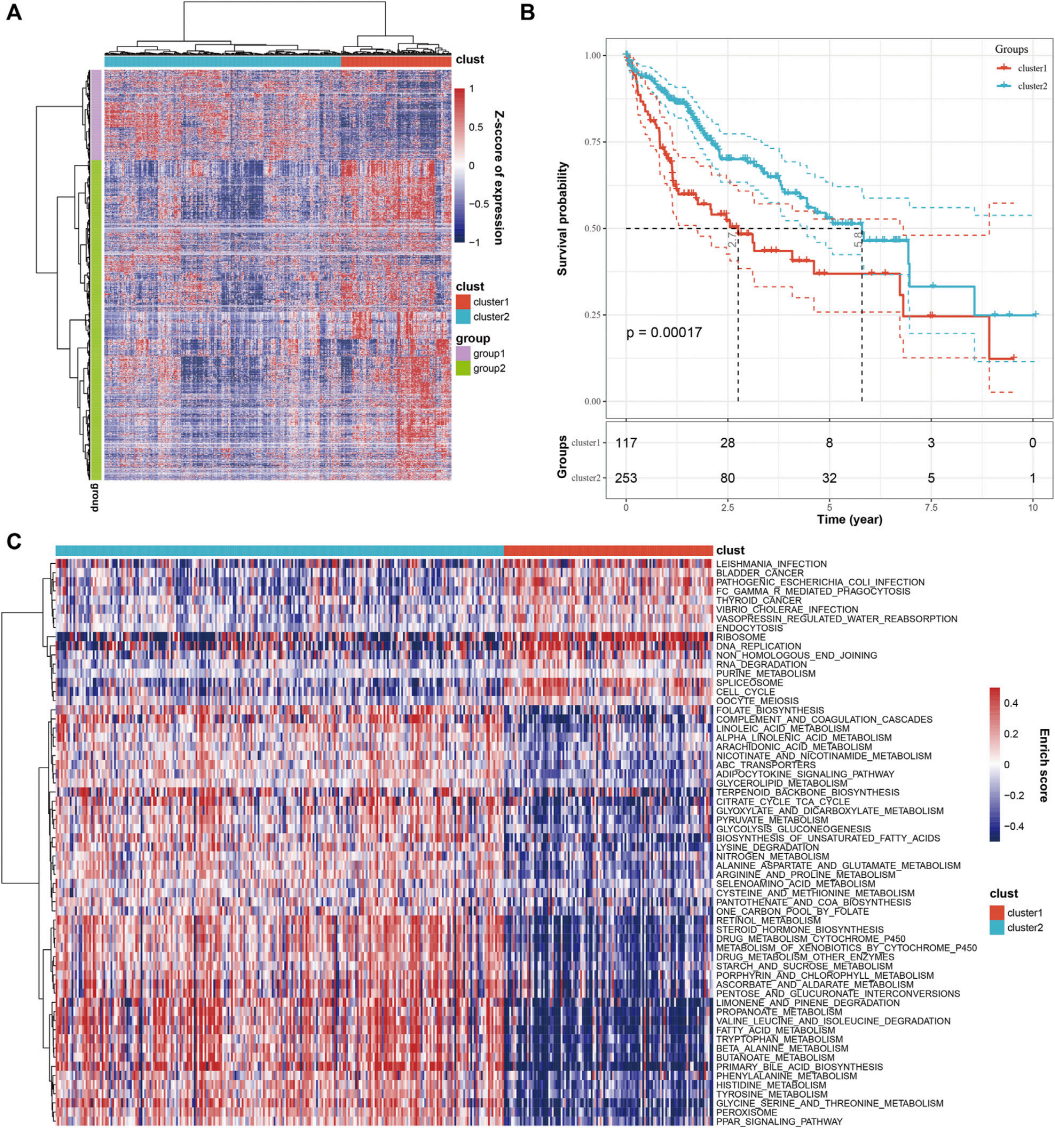
Combined analysis of cells and marker genes from single-cell RNA sequencing with TCGA database. **(A)** The heatmap shows that HCC samples were divided into two clusters based on marker gene expression. **(B)** KM analysis showed that HCC patients within the two clusters exhibited significant differences in OS. **(C)** The comparison of molecular function between the two clusters.

To classify the HCC patients from a molecular perspective, we combined the 2,038 genes and 16,880 cells from the single-cell RNA sequencing screening with the expression spectrum matrix of the TCGA database, which yielded 1,930 genes. Then, we applied unsupervised clustering to the 1,930 genes and the 371 HCC samples, which divided the 371 samples into two molecular clusters, clusters 1 and 2 (C1 and C2) (Figure 2A). Furthermore, a survival analysis of the two clusters was conducted. The KM plot revealed that the C2 patients had a better prognosis than C1 patients (Figure 2B). Next, we performed GSVA analysis to compare the molecular function of both clusters. We found that C1 was mainly associated with cell proliferation signaling pathways, such as cell cycle and DNA replication, while C2 was mainly related to metabolic signaling, including drug metabolism (Figure 2C).

### Immune Infiltration, Signaling Function, Clinical Features, and Therapy of Molecular Clusters

**FIGURE 3:**
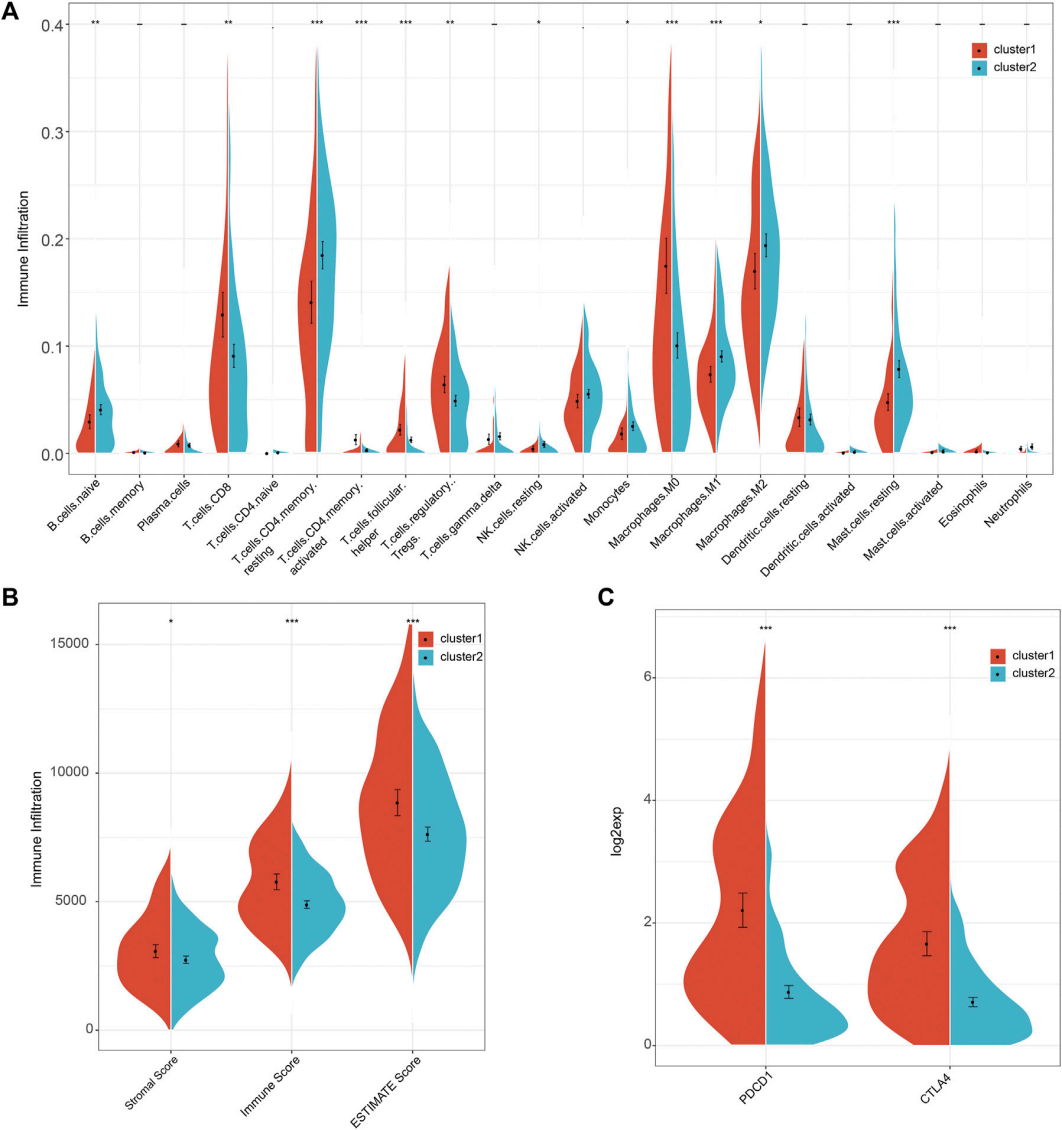
The comparison of immune cell infiltration, immune score, and immunotherapy targets between the two clusters. **(A)** The difference in the infiltration of 22 immune cells between the two clusters. **(B)** The comparison of immune scores between the two clusters. **(C)** The comparison of immunotherapy targets response between the two clusters.

It is widely acknowledged that tumorigenesis in HCC is closely related to the composition of immune cells in the tumor immune microenvironment. Thus, we investigated the composition of immune cells in the two molecular subtypes using CIBERSORT. Results showed that C1 predominantly consisted of follicular helper T cells, regulatory T cells Tregs, and M0 Macrophages, characteristic of the primary immune response. In contrast, C2 was associated with high levels of activated CD4 T memory cells, activated NK cells, Macrophages M2 and M1, and resting Mast cells, characteristic of immune activation during the secondary immune response (Figure 3A). The above results suggested that patients with molecular cluster C1 only exhibited the primary immune response stage, accounting for the poor prognosis, while C2 was at an immune activation stage, related to the activation of immune mechanisms to inhibit tumor cell proliferation, explaining the relatively good prognosis. Next, we calculated the immunity score between the two clusters using the ESTIMATE algorithm. The results showed that C1 had higher immunity scores (Stromal Score, Immune Score, and ESTIMATE Score) than C2 (Figure 3B). Furthermore, we calculated the scores of immunotherapy targets, including PD-L1 and CTLA-4, in both clusters. C1 scores for PD-L1 and CTLA-4 were higher than for C2 (Figure 3C). These results suggested that the high immune infiltration in C1 was associated with poor patient prognosis, suggesting that this particular patient population should be treated with immunotherapy, especially with anti-PD-L1 and CTLA-4 inhibitors. Next, the 1,930 genes screened by single-cell RNA sequencing underwent GSEA. C1 was associated with cell proliferation signaling pathways, while the biosynthesis and metabolism signaling pathways were enriched in C2 (Supplementary Figure S4). These results were consistent with the findings of the GSVA analysis.

**FIGURE 4:**
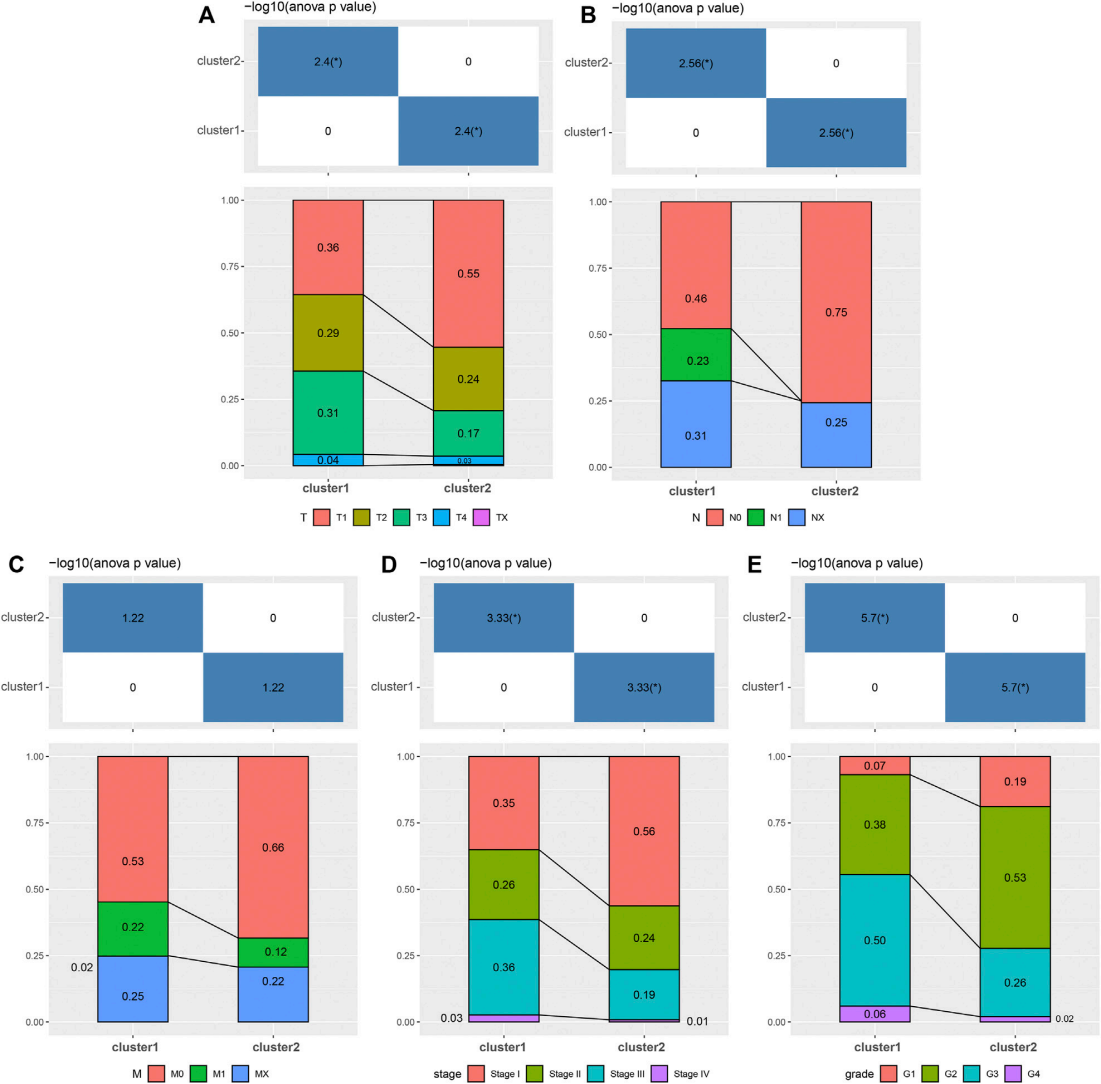
Associated analyses of 2 clusters with clinical features. The distribution of clinical features, including **(A)** T, **(B)** N, **(C)** M, **(D)** stage, and **(E)** grade between 2 clusters.

Moreover, we analyzed the differences in clinicopathologic signatures between both clusters. There were significant differences in T (tumor) (Figure 4A), N (node) (Figure 4B), stage (Figure 4D), and grade (Figure 4E) between the 2 clusters. Among them, C2 correlated with a low pathological grade phase, while C2 was mainly enriched in the low pathological phase of M (metastasis) (Figure 4C), although no statistical significance was found. Furthermore, we analyzed the differences in response to immunotherapy and chemotherapy between both clusters and found that C2 was more sensitive to C1 (Supplementary Figure S5). Our results suggested differences in prognosis between C1 and C2, which could be attributed to differences in immune function, molecular pathways, and clinicopathological signatures.

### Screening of Key Genes From Both Clusters and Analysis of Correlation With Clinical Features

**FIGURE 5:**
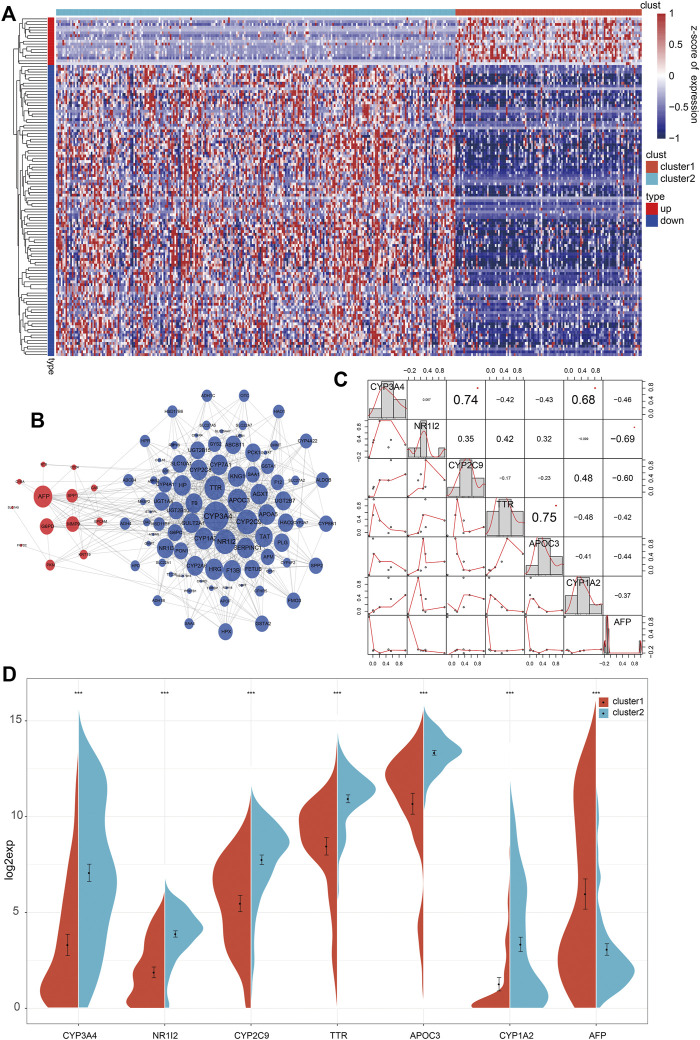
Screening of differentially expressed genes between 2 clusters. **(A)** The heatmap of 121 differentially expressed genes between 2 clusters. **(B)** In the integrated network of 121 differentially expressed genes, red and blue dots represented up-and down-regulated genes. **(C)** The associated analysis of interaction across the seven key genes. **(D)** The expression level of seven genes between 2 clusters.

Based on the significant differences in prognosis, immune function, molecular pathways, and clinicopathologic signatures between both clusters, a total of 121 DEGs were screened (fold change >2), among which 17 genes were upregulated, and 104 genes were downregulated (Figure 5A). Then, 121 key genes were used for correlation analysis and constructing a PPI network. Seven genes (CYP3A4, NR1I2, CYP2C9, TTR, APOC3, CYP1A2, and AFP) with a connective degree of more than 20 were screened out, and the generated PPI network showed that the seven genes were in the center and are closely related to other genes (Figure 5B). Furthermore, we investigated the relationship among the seven genes and found that these seven genes could be divided into three groups (Figure 5C). The first group consisted of three positively correlated genes, CYP3A4, CYP2C9, and CYP1A2, which negatively correlated with the other four genes. It has been established that CYP3A4, CYP2C9, and CYP1A2 are cytochrome P450 family members mainly involved in the metabolic clearance of clinical drugs [11]. The second group consisted of three positively correlated genes (NR1I2, TTR, and APOC3) that negatively correlated with the other four genes. Molecular function analysis indicated that NR1I2, TTR, and APOC3 were involved in transcription, translation, and epigenetics of the genome [12–14]. The third group consisted of only AFP, an oncogene of HCC that exhibited a negative correlation with the six other genes.

**FIGURE 6:**
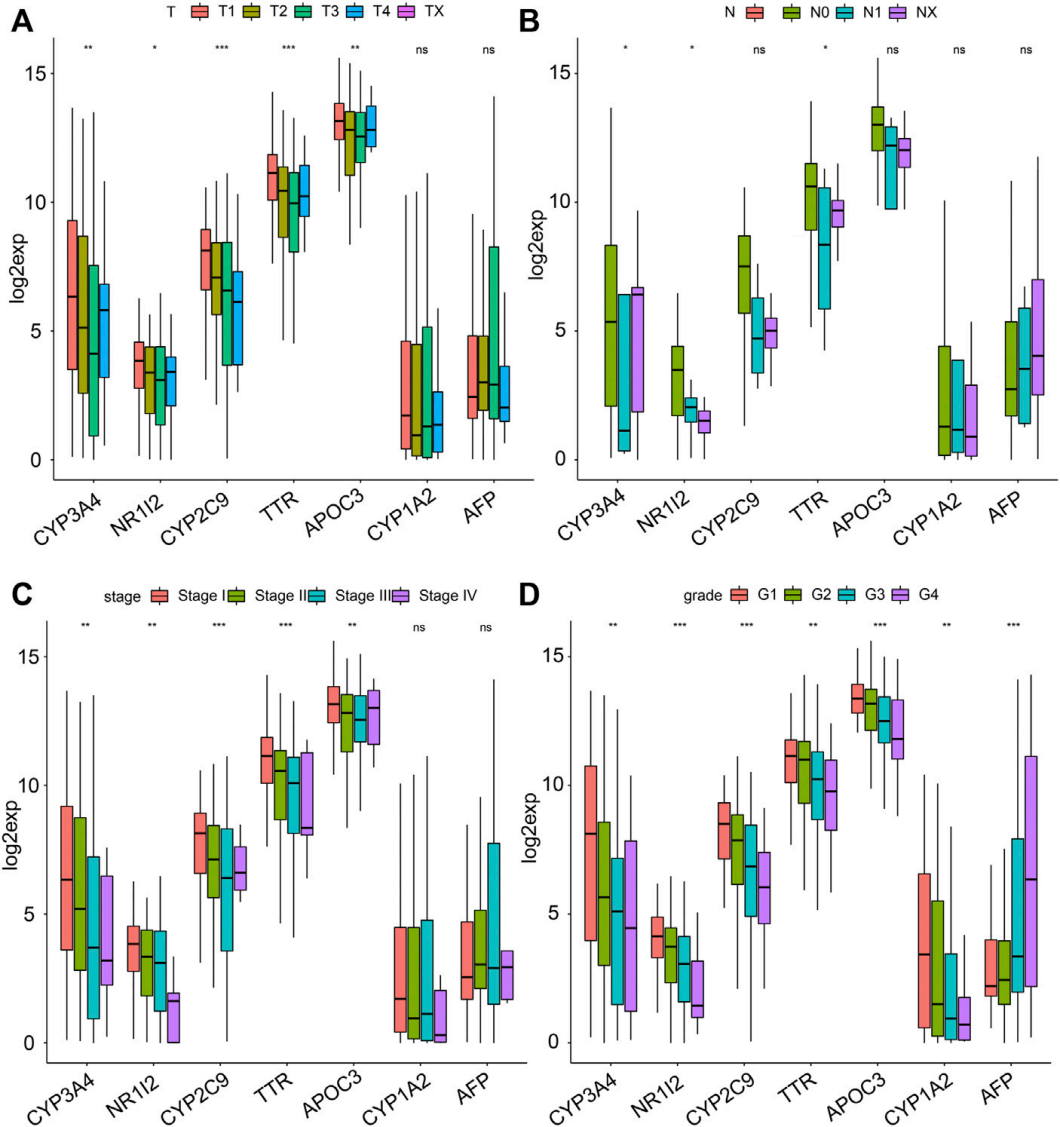
The relationship between the seven genes and clinical features. The distribution of the expression of the seven genes when stratified by **(A)** T, **(B)** N, **(C)** stage, and **(D)** grade.

Since there were significant clinical differences between both clusters and seven genes obtained from DEGs, we further studied the clinical differences associated with the seven genes between the 2 clusters. Analysis of the expression of the seven genes in both clusters showed that in addition to the downregulated expression of APF in C1, the other six genes showed consistency and higher expression in C2 than in C1 (Figure 5D). Then, we analyzed the clinicopathologic signatures, including stage, T, N, and M. Overall, the results revealed that AFP was positively correlated with clinicopathologic progression, while the other six genes were negatively correlated (Figures 6A–D). KM analysis of the seven genes showed that high expression levels of CYP3A4, NR1I2, CYP2C9, and APOC3 were associated with better survival. Patients with high AFP expression had a worse prognosis than patients with low expression levels, although no statistical significance was found (Supplementary Figure S6A). The above results proved that our molecular classification (C1 and C2) based on single-cell sequencing and data mining has clinical variability and indicated that the seven key genes reported were significant biomarkers. In C1 patients, high expression of oncogene AFP was associated with poor prognosis and clinicopathological progression, while the other six genes were associated with a favorable prognosis and low clinicopathological stage.

### Construction of Prognostic Model and Validation

**FIGURE 7:**
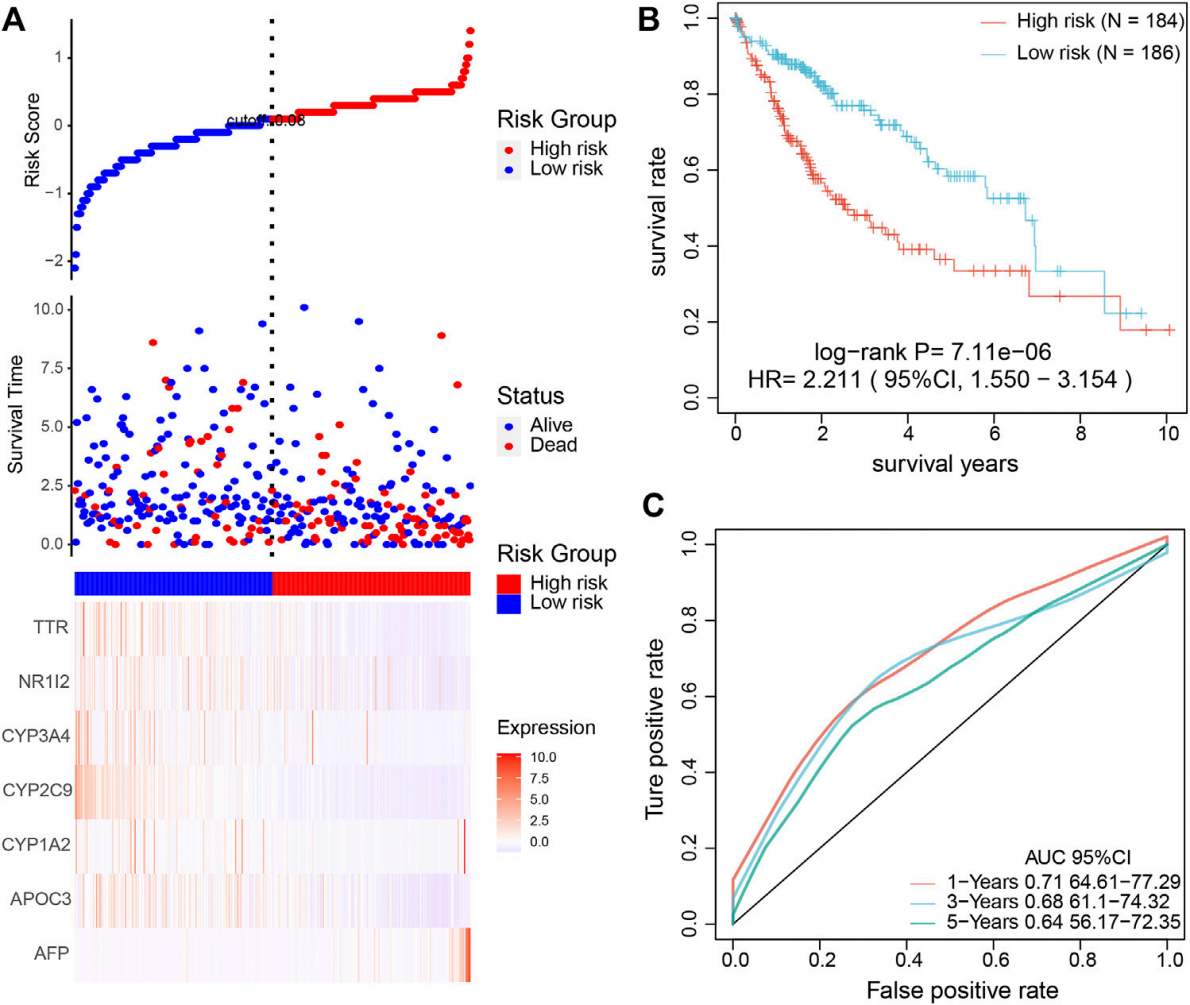
Validation of the prognostic model to determine its clinical predictive value. **(A)** The association among risk-score of the prognostic model, survival time, survival state, and the expression of the seven key genes. **(B)** KM analysis of HCC patients based on high and low risk. **(C)** The sensitive prediction of the prognostic model was assessed by ROC curve analysis.

The seven key genes were selected for UniCox and LASSO Cox regression analysis to construct a prediction model. These seven genes were used as a signature to predict the OS of patients. Then, we calculated the risk score of each patient according to the expression level in the samples and plotted the risk-score distribution. As shown in Figure 7A, the OS of samples with a high risk score was significantly shorter than samples with a low risk score, suggesting that the high risk score sample had a worse prognosis. KM demonstrated that patients with a high risk score have a shorter survival time than those with a low risk score (Figure 7B). To estimate the sensitivity of the prognostic model based on the seven genes, ROC curves were drawn. The under the curve (AUC) for survival at 1, 3, and 5 years were 0.71, 0.68, and 0.64, respectively (Figure 7C). Correlation analysis of high/low risk patients and treatment outcomes indicated that low risk patients were more sensitive to immunotherapy and chemotherapy than high risk patients. Next, we conducted a time-dependent ROC curve analysis to characterize the predictive potential of the prognostic model, TNM, age, and the combination of all existing features. The results showed that the AUC of the prognostic model (0.69) was higher than the AUC for TNM (0.64) and age (0.53) (Supplementary Figure S8A). As expected, combining our prognostic model with TNM staging and age improved our ability to predict prognosis (AUC = 0.73). The nomogram showed that the risk score based on the prognostic model had the longest line, indicating it exerted the most significant influence on the prediction of survival rate and that the prognostic model contributed the highest number of risk points (from 0 to 100) than other clinical features (Supplementary Figure S8B). The calibration curves for the nomogram showed good agreement between predicted outcomes at one, three, and 5 years and the actual outcomes (Supplementary Figure S8C). Moreover, to identify the independence of the prognostic model based on the seven key genes for clinical application, we used univariate and multivariate Cox regression to analyze the HR, 95% CI of HR, and *p*-value. Univariate Cox regression analysis showed that the prognostic model, T, N, M, and TNM were significantly correlated with survival. The risk score for HR based on the prognosis was the highest among all clinical features (Supplementary Figure S9A). The multivariate COX regression analysis showed that only the prognostic model was significantly correlated with survival, with the highest risk score among all clinical features (Supplementary Figure S9B).

To further validate the accuracy of the prognostic model, we used another independent database. As expected, the OS of samples with a high and low risk score and ROC and KM analysis findings (Supplementary Figure S10) were consistent with TCGA database analysis results (Figure 7). In addition, we conducted a multivariate Cox analysis to compare the prognostic model with four published models ([15–18]) using the C-index. The prognostic model in our study achieved a higher C-index than the other four published models (Supplementary Figure S11). The above results suggested that the prognostic model based on the seven key genes from single-cell sequencing could effectively predict the prognosis for HCC patients.

## Discussion

Intratumoral heterogeneity poses a significant challenge for the clinical management of HCC [4]. With the rapid development of genomics in cancer, it is now possible to explore the variation at the genomic level and screen biomarkers closely related to the pathogenesis of HCC [7]. However, important genes can be missed when dealing with large numbers of transcriptome analyses of cell populations, and most screening studies have predominantly aimed to distinguish tumor cells from non-tumor cells. This may explain why HCC patients gradually develop drug resistance after targeted therapy for a certain period [8, 19]. Importantly, single-cell sequencing provides an innovative approach to studying the tumor microenvironment and intracellular heterogeneity of HCC by analyzing the transcriptome of thousands of individual cells [9]. In contrast with previously conducted studies, in the present study, single-cell data was used to obtain accurate cells and genes and integrated with conventional sequencing data for comprehensive analysis and research. Subsequently, the obtained molecular typing was compared and analyzed, and the prognostic model was constructed. The prognostic model was associated with clinical information, compared with published models, and verified using independent data. Importantly, our model overcomes limitations of tumor heterogeneity and provides a multi-dimensional approach to obtain accurate targets and a more efficient clinical model. Overall, our study mined HCC targets based on conventional sequencing data at the single-cell level, which provided an effective model for accurate diagnosis and treatment of HCC.

In this study, using single-cell RNA sequencing, we characterized the features of various cell types and corresponding tumor marker genes and screened significant cells and genes using bioinformatics analysis methods. Combined with gene expression datasets, the identified significant cells and genes were used to divide HCC patients into 2 clusters with distinct clinical characteristics, immune function, and signaling pathways. We then developed a robust prognostic model based on key genes identified from single-cell RNA-sequencing and analyzed the relationship with clinical characteristics to validate its clinical value. Overall, our study refined our understanding of the cellular composition characteristics and molecular signature of HCC cells and provided novel targets for clinical application.

In the present study, the single-cell RNA-sequence profiling of HCC cells was subjected to strict quality control, and 15,093 cells with high variability were screened for clustering analysis. To obtain a precise molecular classification of HCC, cell lines with high levels of homology must be obtained. Therefore, we investigated the categorizations and proportions of cell types in all 20 sub-clusters and found that HCC cells represented 97% of the 15,093 cells, suggesting high single-cell homology in our study. Such high purity tumor cells are important for further accurate molecular classification and target mining. It is widely acknowledged that tumor tissue consists of many structures, and tumor tissue components can be divided into parenchyma and stroma [20]. It is well-established that the interstitial tumor tissue, including immune tissues, fiber tissues, and blood vessels, must be removed to obtain tumor parenchymal cells and reduce the interference of external factors during the study of tumor gene expression and epigenetic modification [20]. Unlike previous studies where cells and genes were collected from bulk transcriptomics [21, 22], the high purity tumor cells (*n* = 15,093) corresponding to 2,038 genes were subjected to a combination of bulk sequencing and correlation analysis with clinical information to yield a molecular classification of HCC in the present study. We found that the HCC patients could be divided into 2 clusters with significant differences in clinical features, immune function, and signaling pathways. Importantly, our approach provides more accurate molecular classification and data mining for HCC biomarkers.

Analysis of the infiltration of immune cells between the two clusters showed a higher infiltration of follicular helper T cells, Tregs, and M0 Macrophages in C1, characteristic of the primary immune response. It has been established that cancer-related immune cells can recognize, attack and kill malignant tumor cells, producing a rapid immune response and playing a long-term anticancer role [23]. The antitumor immunity can reportedly activate the immune defense system and the immune cells [24]. We hypothesized that C1, associated with an unfavorable prognosis, resulted from the primary immune response of resting immune cells. In contrast, C2 was associated with high levels of activated CD4 memory T cells, activated NK cells, and M2 and M1 macrophages, suggesting an activated immune state. An increasing body of evidence suggests that the activation of immune cells, such as CD4, CD8, and NK cells, can kill tumor cells via molecular mechanisms [25, 26]. The good prognosis of C2 may be attributed to the activation of immune system against tumor cells. However, the expression of immunotherapy gene targets of PD-L1 and CTLA-4 in C1 was higher than in C2. Drugs targeting PD-L1 and CTLA-4 have been used in clinical trials for HCC and bring significant benefits to patients [27]. The high expression of PD-L1 and CTLA-4 in C1 suggested that immunotherapy could be an effective therapeutic approach for C1 patients.

Our prognostic model of seven genes derived from single-cell studies exhibited robust prediction efficiency and correlated with clinical characteristics. Both data processing and target screening were obtained from data analysis. Our comprehensive prognostic model was based on the integration of these seven genes, which yielded superior accuracy than these single genes. Alpha-fetoprotein (AFP) is a widely acknowledged diagnostic and prognostic index for HCC and an oncogene that causes HCC [28]. However, AFP cannot be used as a specific target for all HCC patients. Moreover, HCC patients do not always exhibit high AFP levels during clinical practice, while patients with elevated APF do not necessarily suffer from HCC [29]. Our prognostic model consists of seven key genes that exhibited high prognosis prediction efficacy. Further research revealed that the seven key genes could be separated into three groups based on molecular function. The first group consisted of CYP3A4, CYP2C9, and CYP1A2 and was negatively associated with AFP. These three genes are cytochrome P450 family members responsible for more than 90% of the metabolic clearance of clinical drugs [11]. The second group consisting of NR1I2, TTR, and APOC3, was negatively associated with AFP. Overwhelming evidence substantiates these three genes’ molecular functions are transcriptional regulation, epigenetic modification, and protein translation [12–14]. Analysis of individual genes found that high expression of CYP3A4, NR1I2, CYP2C9, and APOC3 was associated with a good prognosis, while high expression of AFP was related to a poor prognosis. However, the seven genes that were included in our prognostic model were risk factors after KM, univariate, and multivariate COX regression analysis. We hypothesized that these seven genes were not independent but their mutual interactions acted as a single intricate prognostic marker. Further study of the regulatory relationships among the seven key genes, may help elucidate their underlying role in the pathogenesis of cancer. In addition, the performance of our prognostic model was superior to clinical features. However, it remains largely unknown whether the prognostic model established is more effective than other clinical indicators at the clinical level, warranting the need for further studies. Indeed, the application of our findings in conjunction with current clinicopathological staging may improve the diagnostic and prognostic evaluation of HCC.

## Conclusion

This study provided a novel approach and the foothold for future in-depth studies and promoted the development of clinical precision medicine for HCC. This study integrated single-cell RNA sequencing and big data sequencing to reveal specific cells and genes from single-cell expression profiles. We found that the HCC could be divided into two clusters with distinct clinical characteristics, immune function, and molecular pathways. Moreover, we established a prognostic model based on seven key genes, which showed high efficiency in predicting the prognosis of HCC. Importantly, this research provided a novel molecular classification of HCC and unraveled biomarkers that could be used clinically.

## Data Availability

The original contributions presented in the study are included in the article/Supplementary Material, further inquiries can be directed to the corresponding authors.

## Supplementary Material

Supplementary Figure S1 The flow chart of this study.

Supplementary Figure S2 Cell screening for single-cell RNA sequencing analysis. Genes were expressed in at least 100 cells, and at least 2,000 genes were detected in each cell. The feature, read count, and percent. mt of each sample cell **(A)** before and **(B)** after filtration. Percent. mt is the proportion of detected mitochondrial genes.

Supplementary Figure S3 Screening for genes with highly variable expression. The red and black dots represent the highly variable and non-variable genes.

Supplementary Figure S4 GSEA signaling functional analysis of 2 clusters. The enrichment of signaling function of **(A)** C1 and **(B)** C2.

Supplementary Figure S5 Differences in sensitivity of molecular subtypes to immunotherapy and chemotherapy for the drug of **(A)** erlotinib, **(B)** sunitinib, **(C)** saracatinib, **(D)** paclitaxel, and **(E)** TAE684.

Supplementary Figure S6 KM analysis of the seven key genes from single-cell RNA sequencing.

Supplementary Figure S7 Differences in sensitivity of high/low-risk patients to immunotherapy and chemotherapy for the drug of **(A)** erlotinib, **(B)** sunitinib, **(C)** saracatinib, **(D)** paclitaxel, and **(E)** TAE684.

Supplementary Figure S8 Validation of the predictive efficiency of the prognostic model by comparison with other clinical features. **(A)** ROC curve was used to characterize the predictive potential of the prognostic model, TNM, age, and the combination of all the existing features. **(B)** Nomogram plots were used to validate the predictive performance of the prognostic model when compared with TNM staging. **(C)** Calibration curves of survival at 1, 3, and 5 years for the nomogram.

Supplementary Figure S9 Forest plots for univariate and multivariate survival analyses with clinical features and prognostic model. The forest plots of **(A)** univariate and **(B)** multivariate survival analyses were used to validate the independence and efficiency of the clinical features and prognostic model.

Supplementary Figure S10 Validation of the prognostic model from an independent database. **(A)** The association among the risk score of the prognostic model, survival time, survival state, and the expression of the seven key genes. **(B)** KM analysis of HCC patients based on high and low risk. **(C)** The sensitive prediction of the prognostic model was assessed by ROC curve analysis.

Supplementary Figure S11 Comparison between the prognostic model and 4 published models using the C-index.
