# Supplementary figures and images for: Identification and Characterization of Genes Related to the Prognosis of Hepatocellular Carcinoma Based on Single-Cell Sequencing
Source: Pathol Oncol Res. 2022 Aug 25;28:1610199. doi: 10.3389/pore.2022.1610199 (PMC9454301; doi:10.3389/pore.2022.1610199)

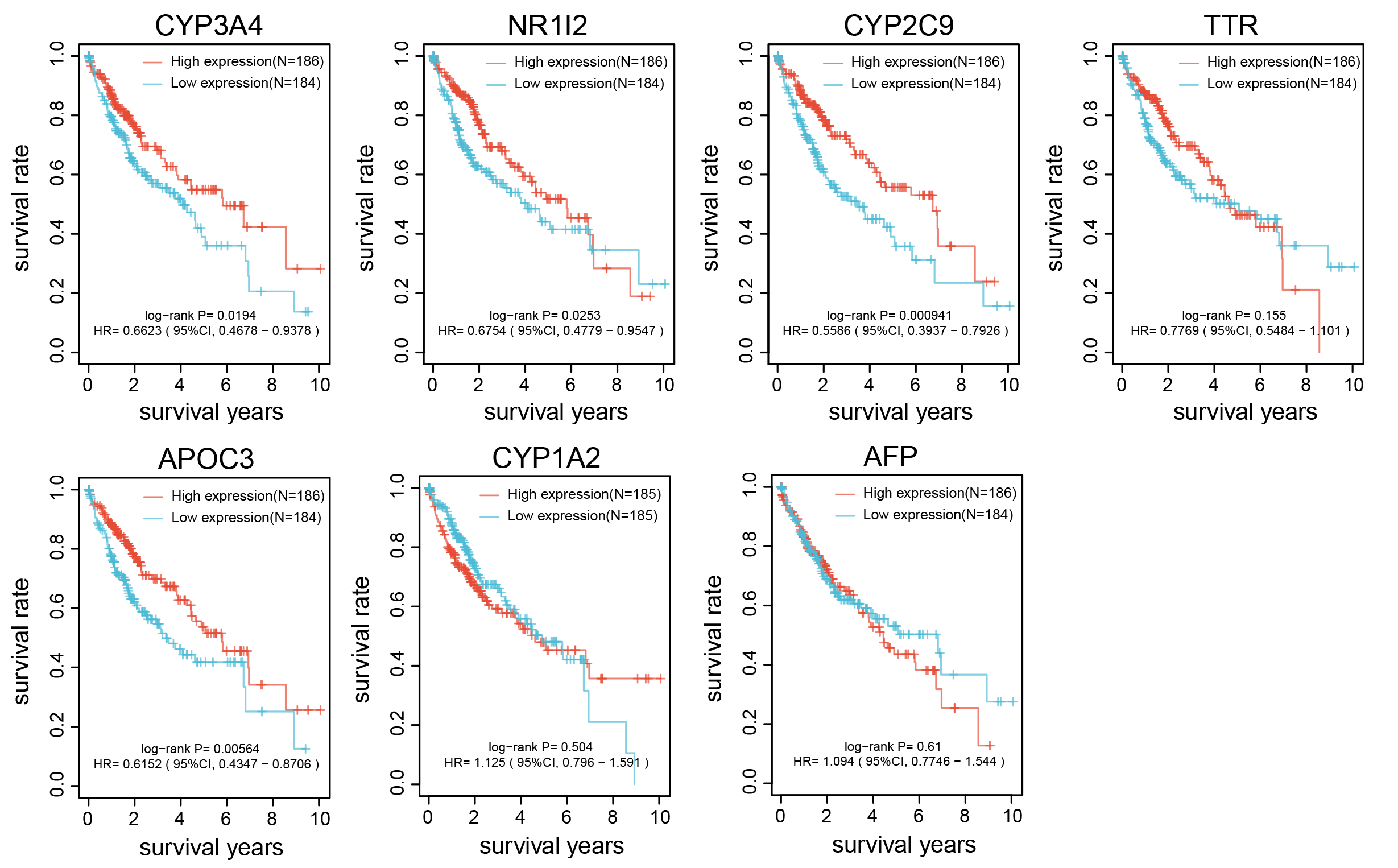

Supplement: Supplementary file 1 [file Image6.tif]

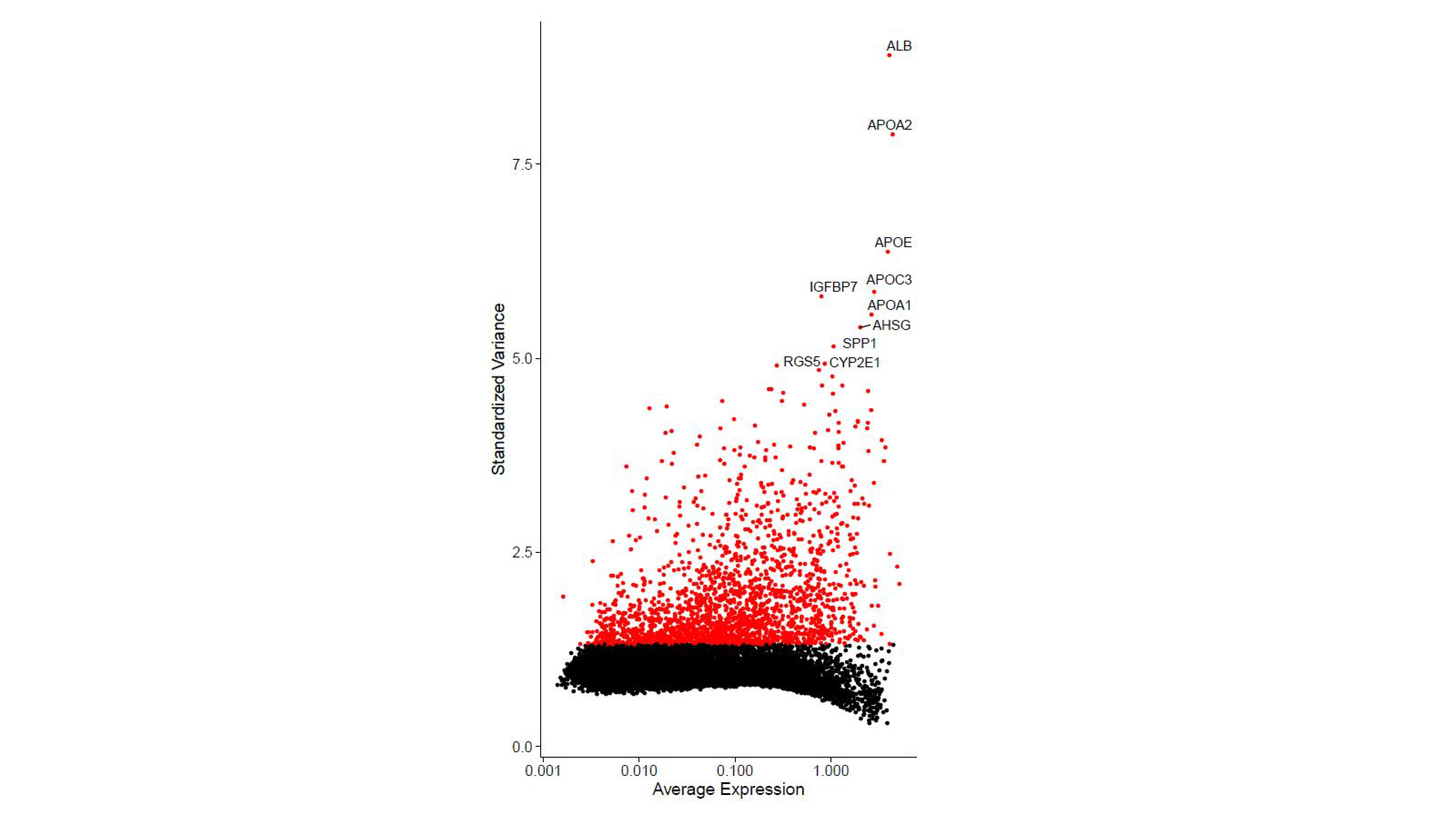

Supplement: Supplementary file 3 [file Image3.TIF]

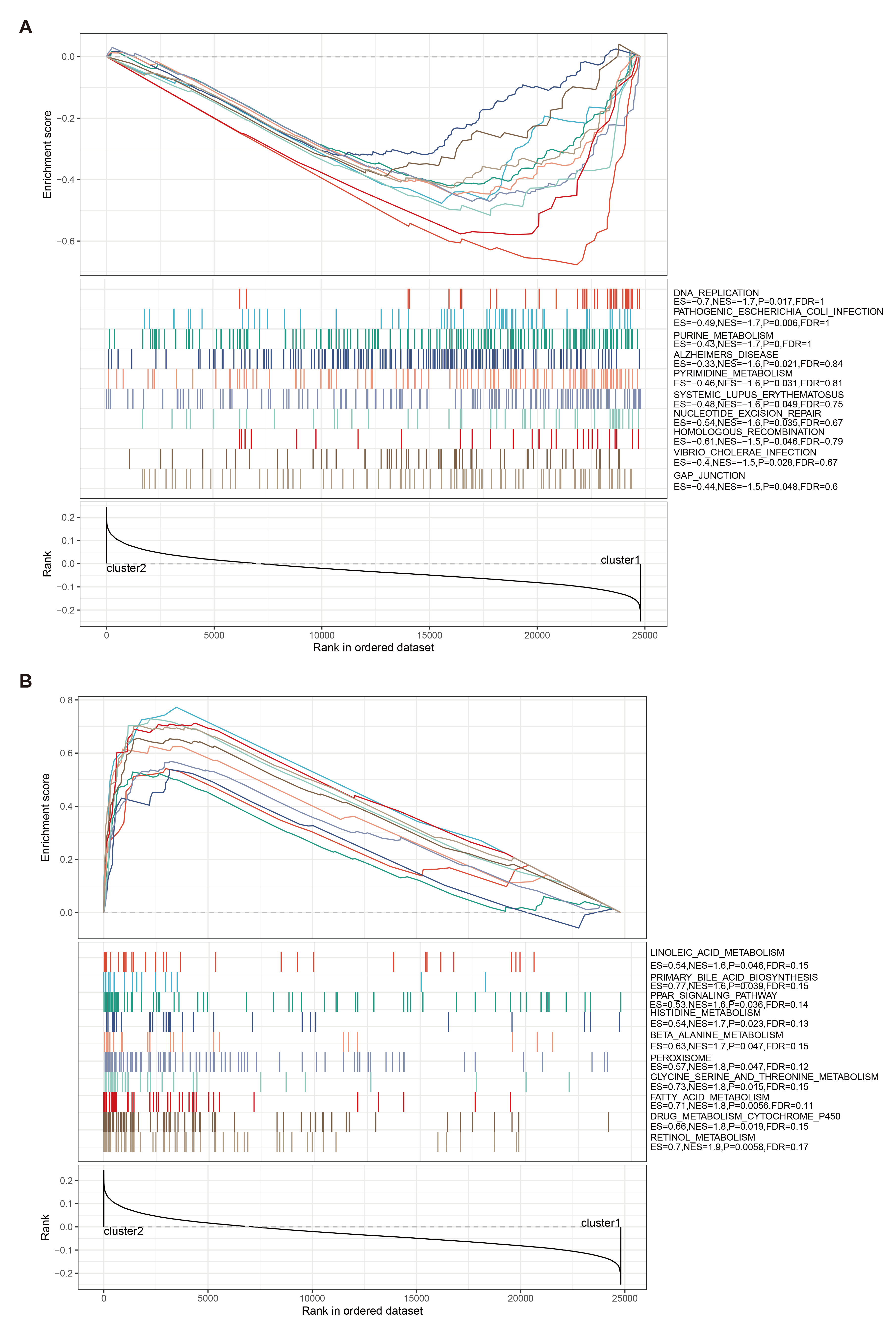

Supplement: Supplementary file 4 [file Image4.TIF]

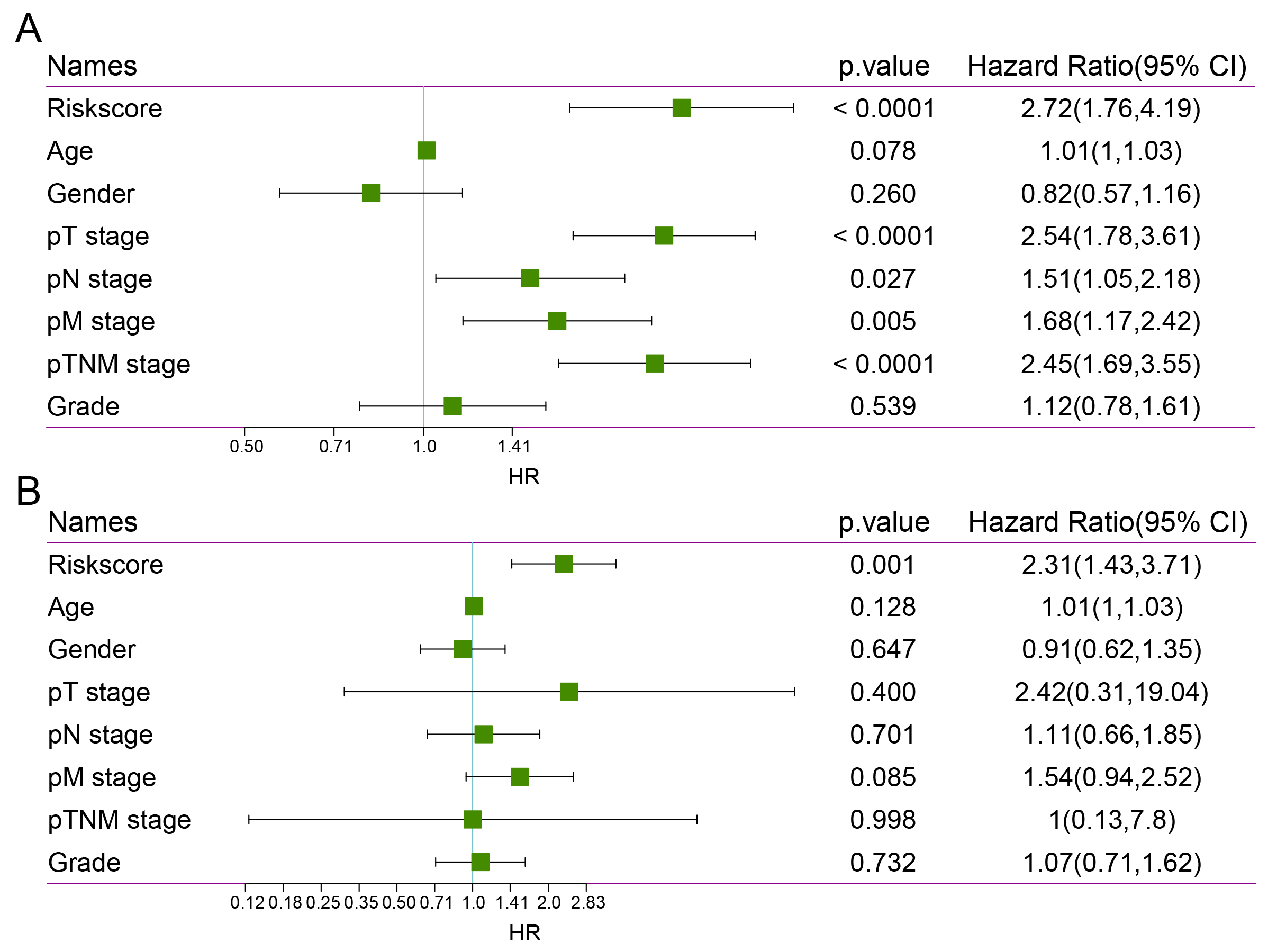

Supplement: Supplementary file 5 [file Image9.TIF]

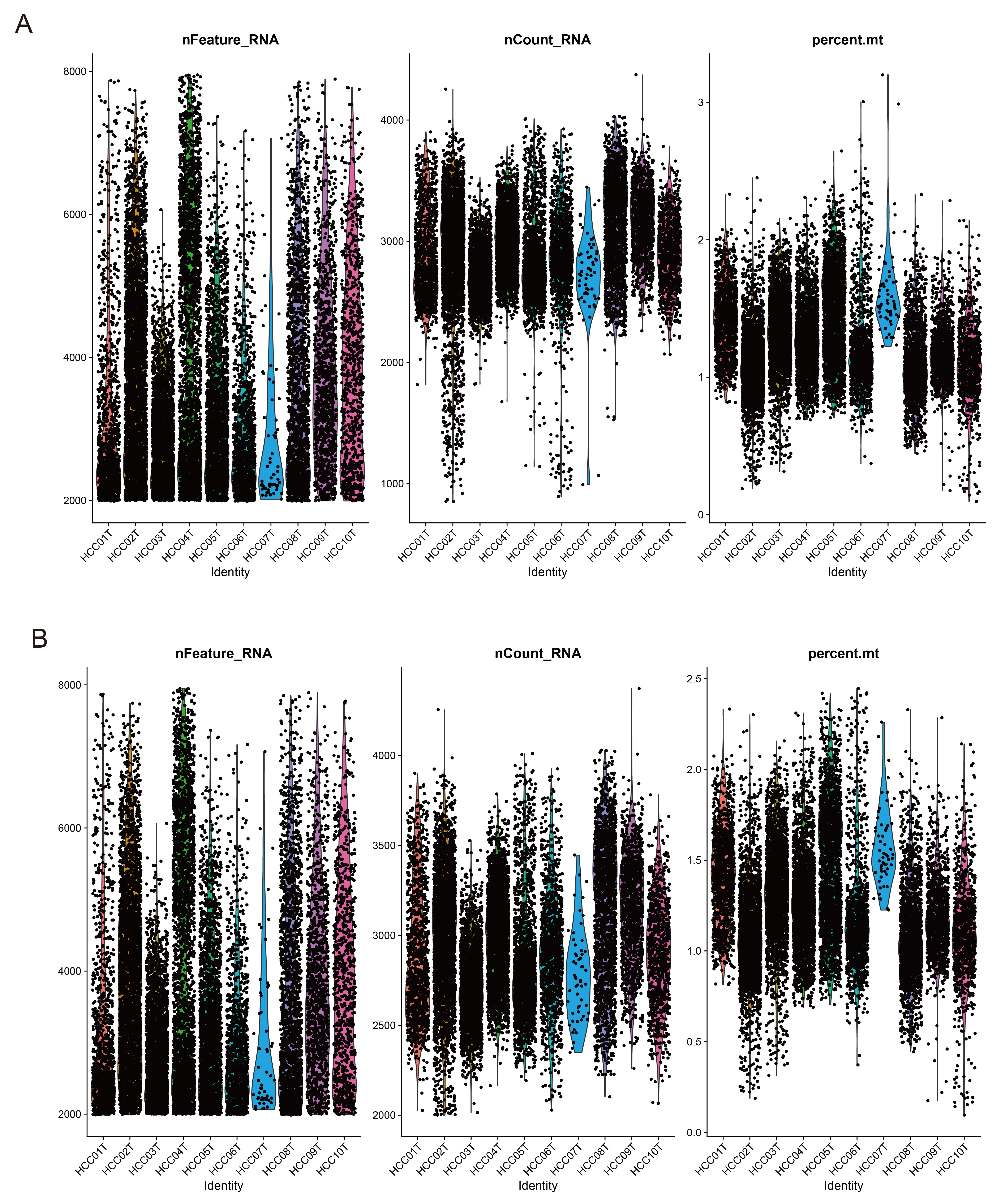

Supplement: Supplementary file 6 [file Image2.TIF]

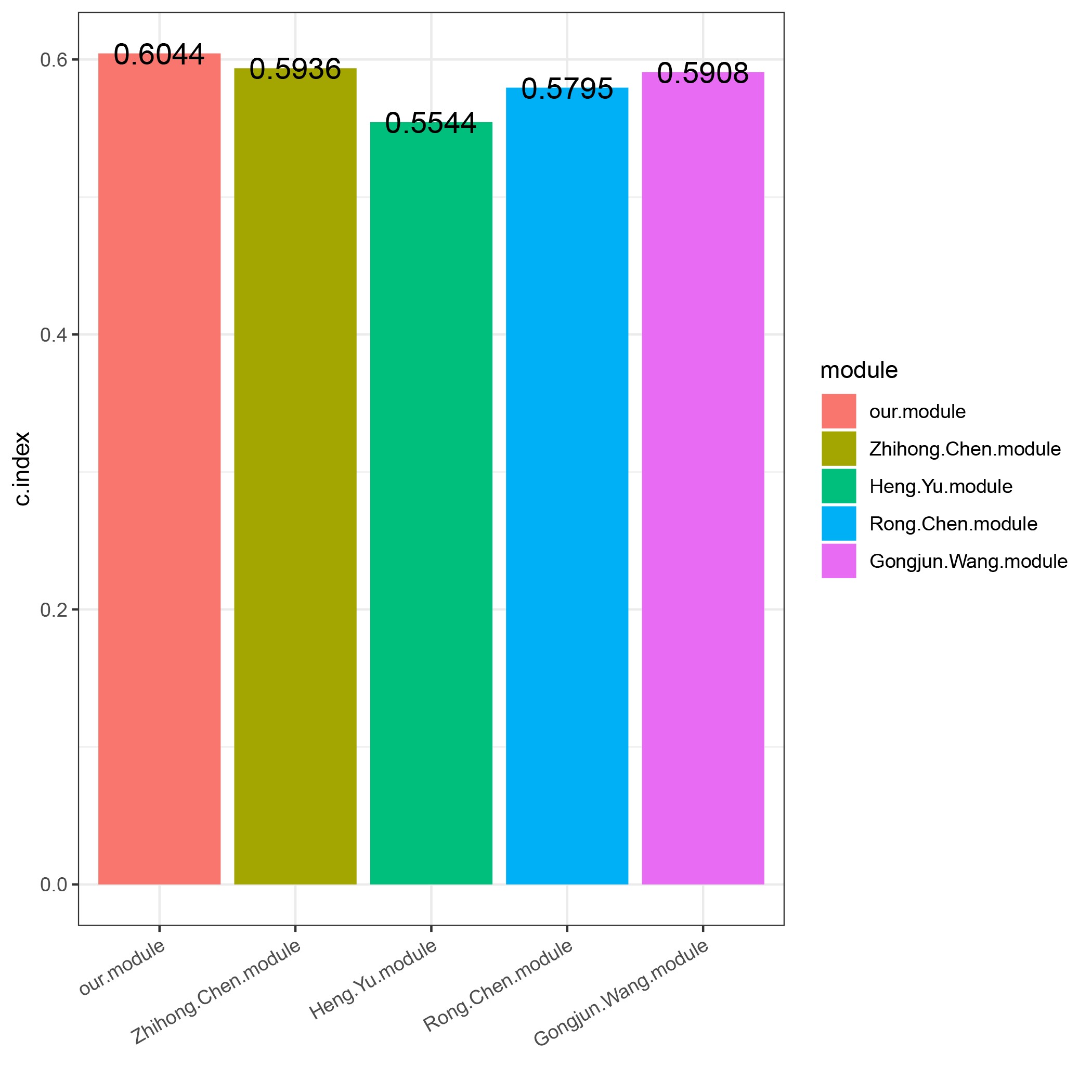

Supplement: Supplementary file 7 [file Image11.TIF]

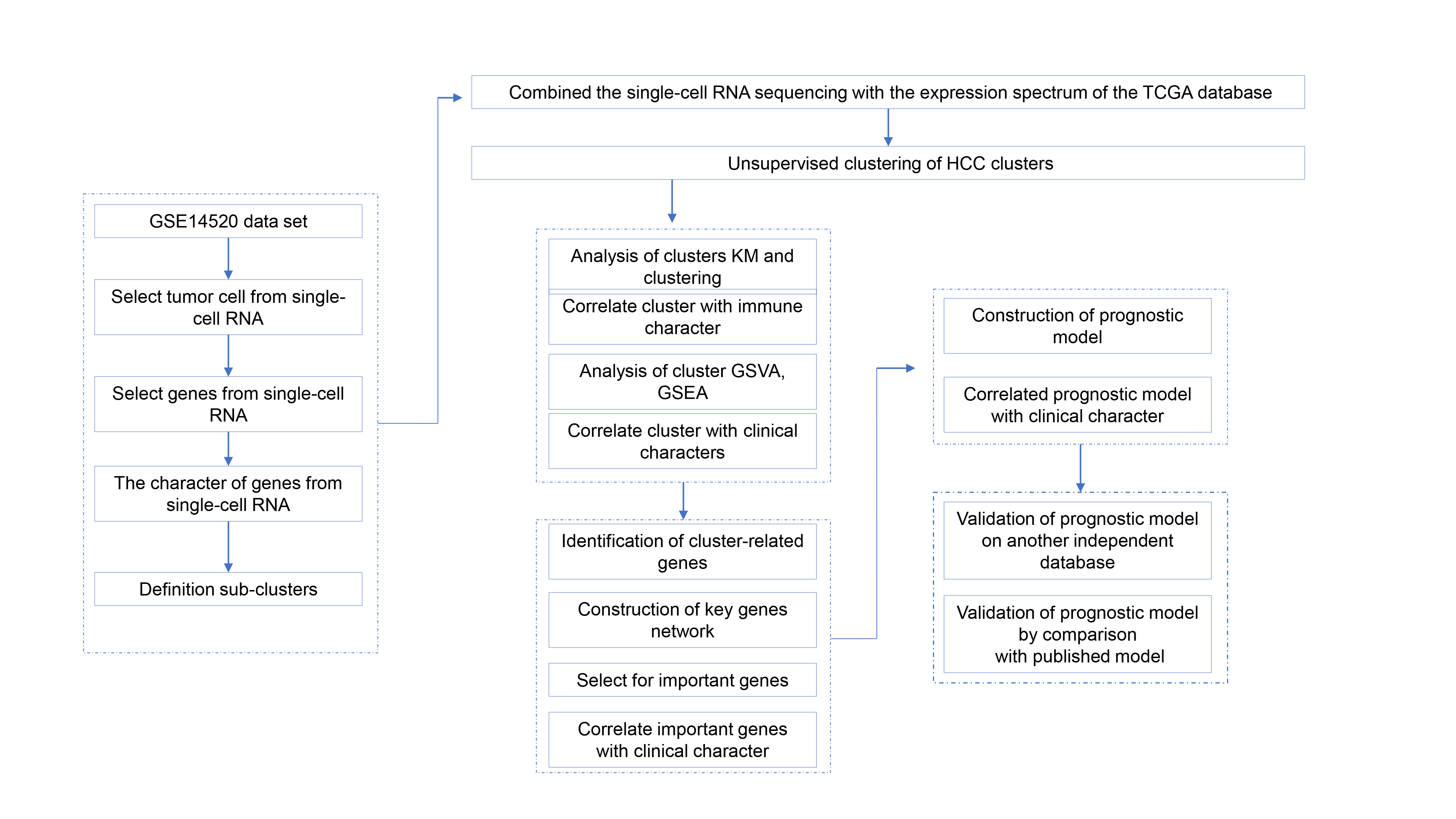

Supplement: Supplementary file 8 [file Image1.TIF]

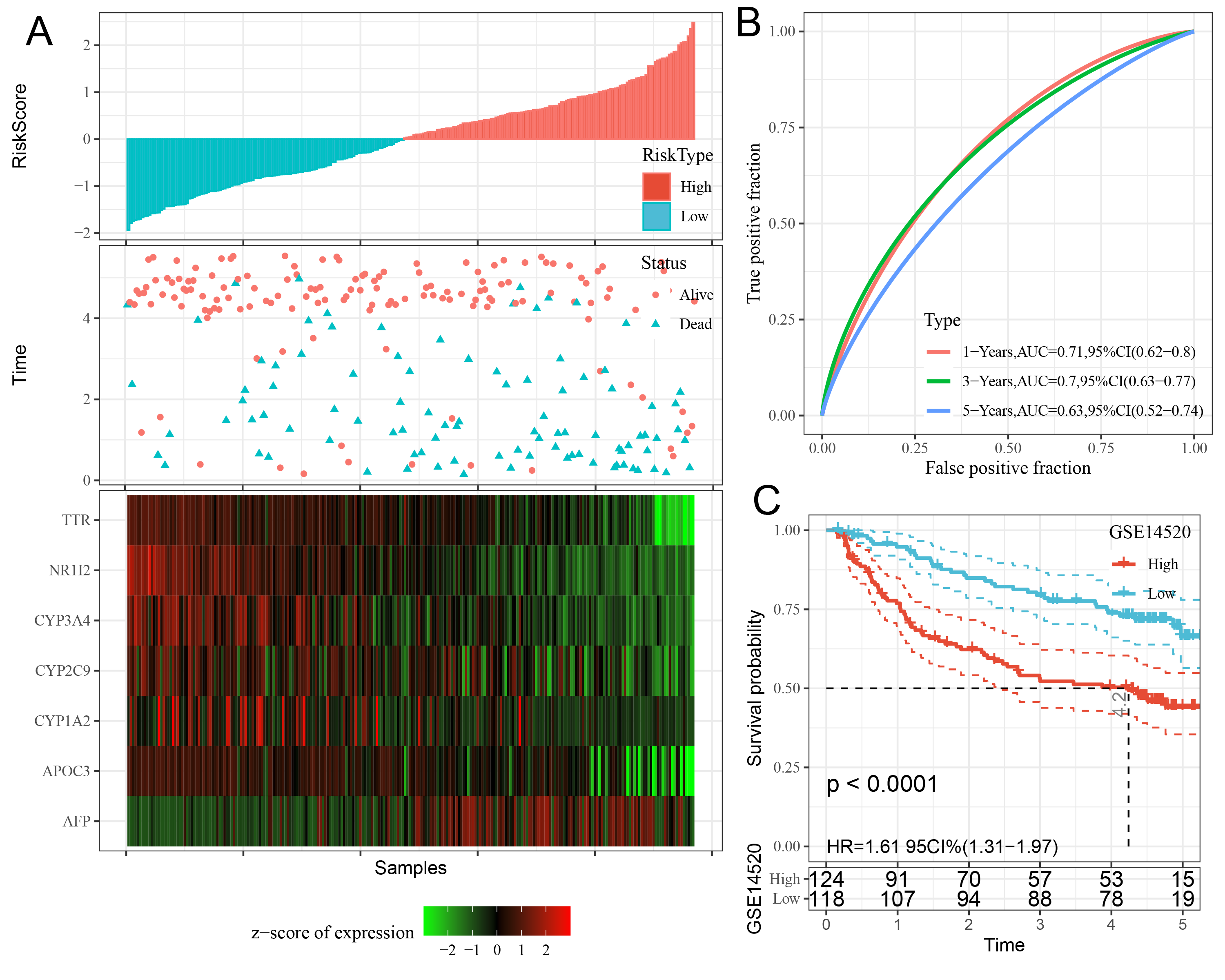

Supplement: Supplementary file 9 [file Image10.TIF]

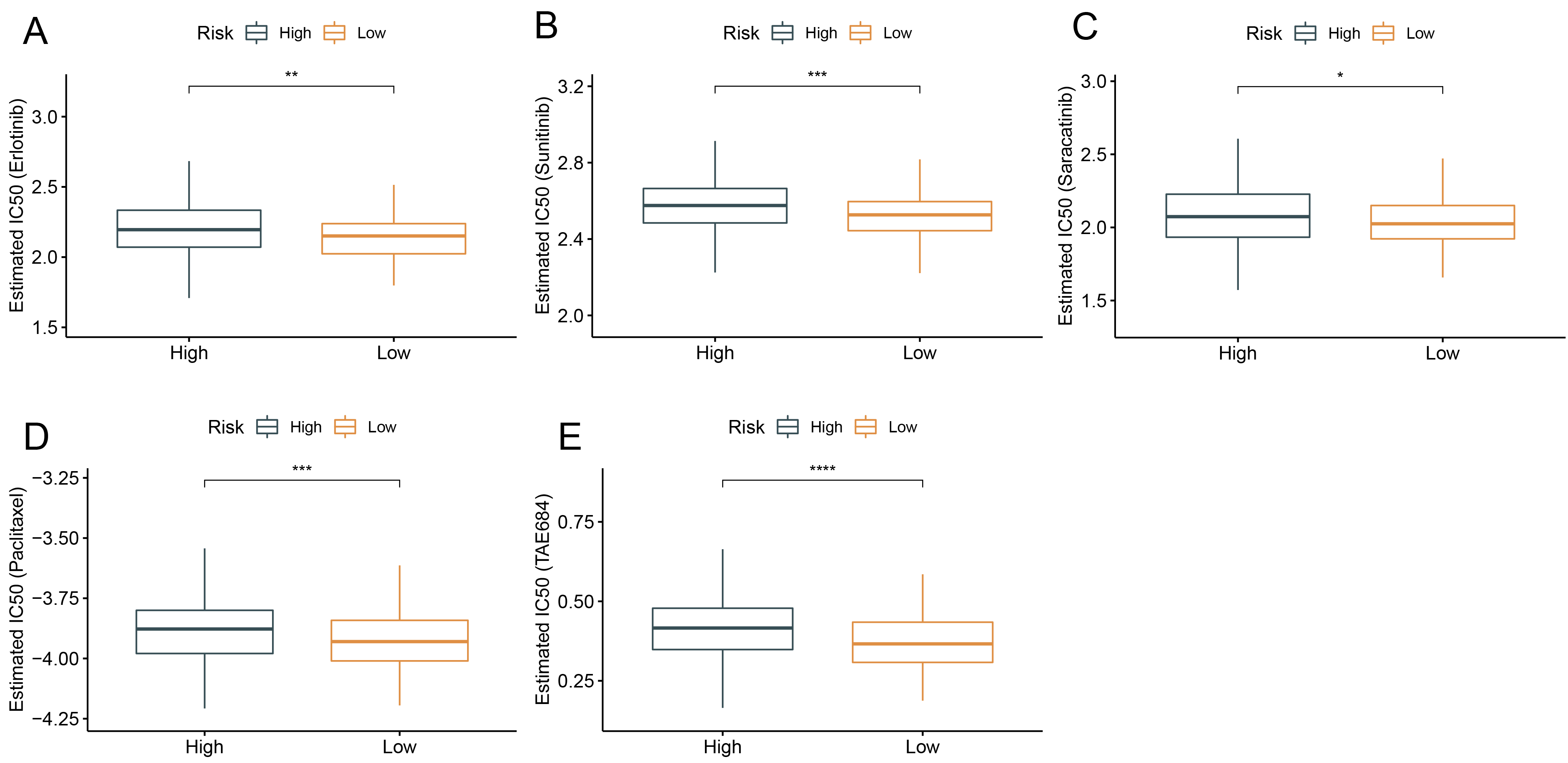

Supplement: Supplementary file 10 [file Image7.TIF]

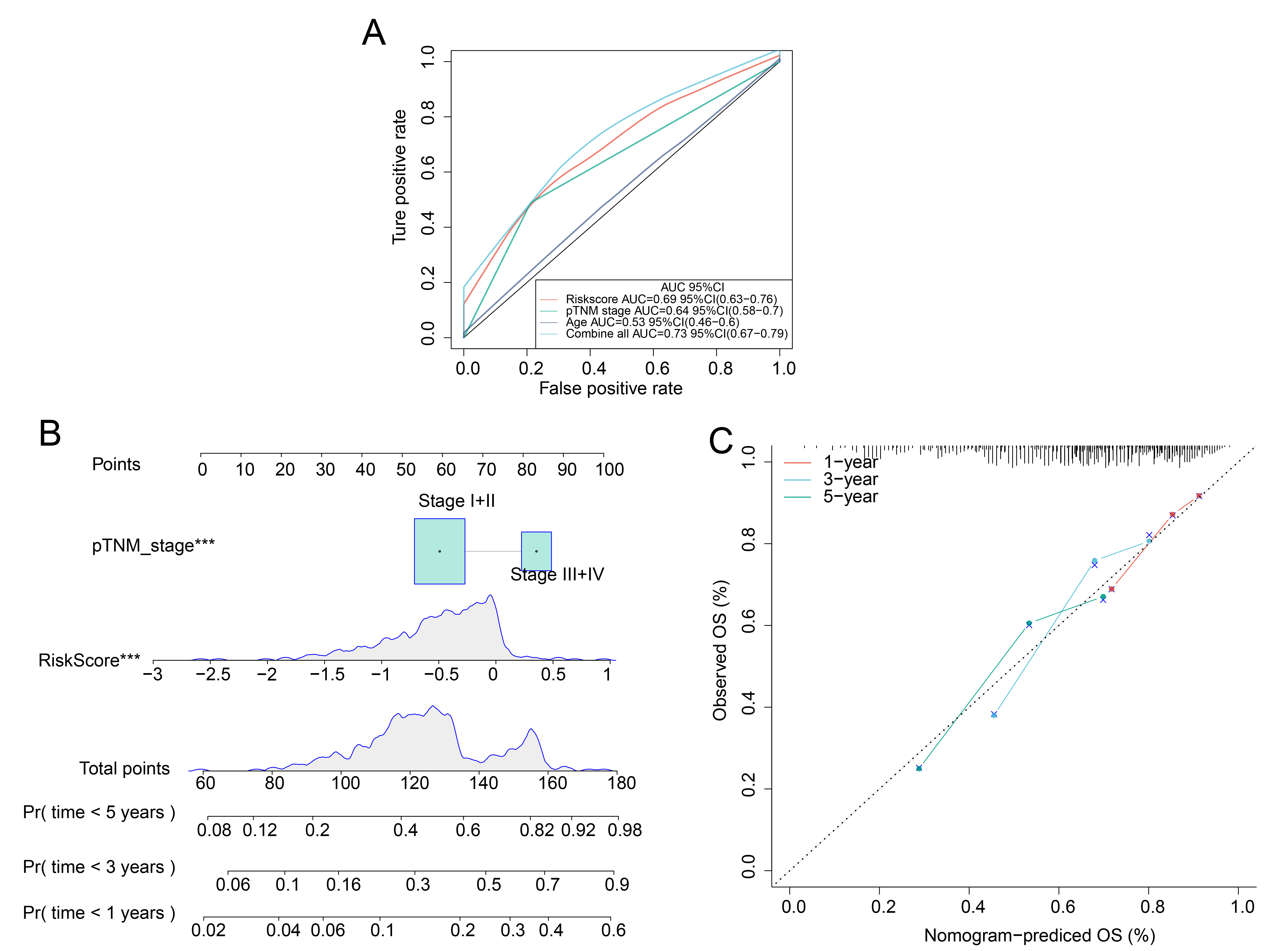

Supplement: Supplementary file 12 [file Image8.TIF]

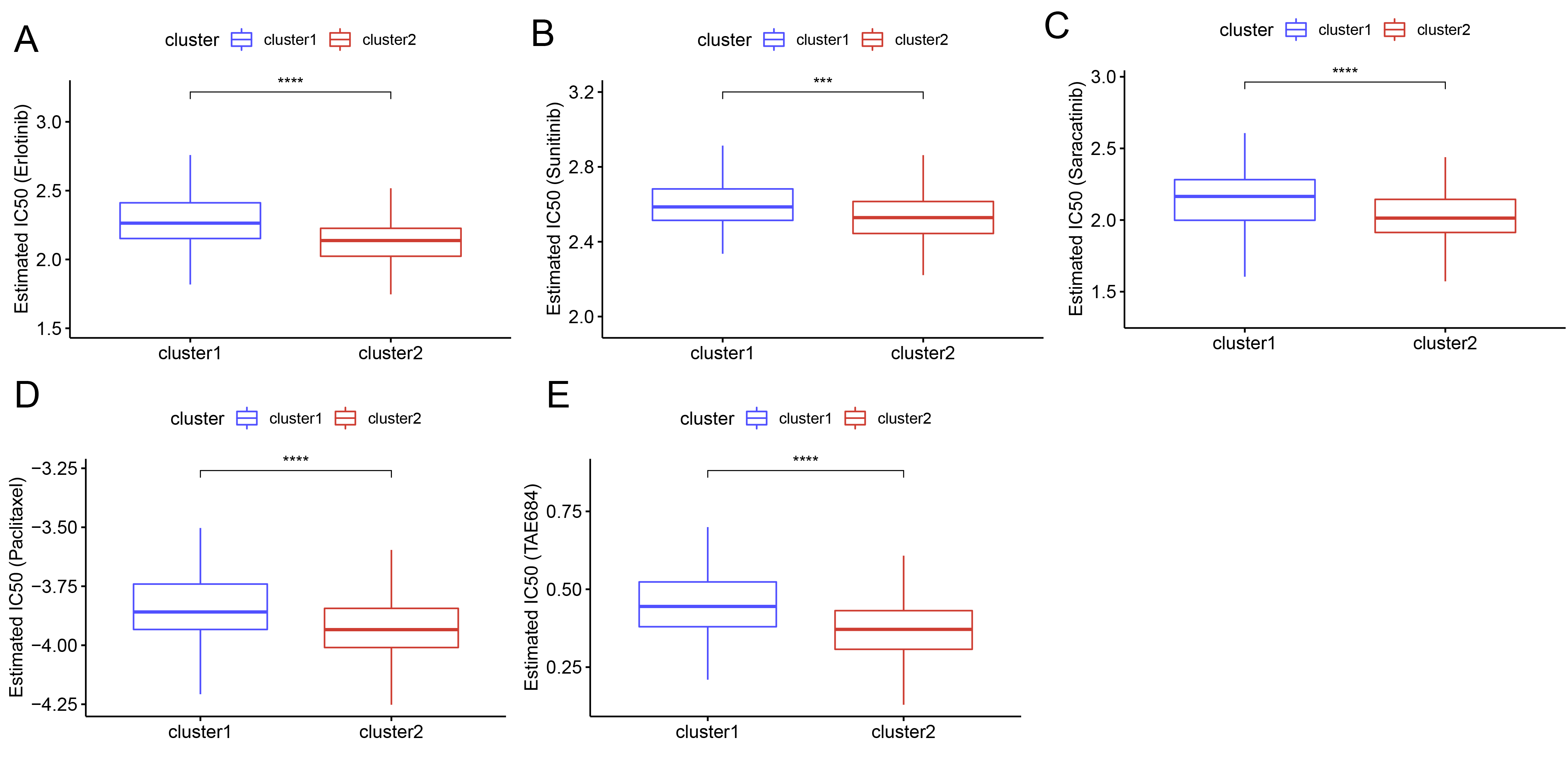

Supplement: Supplementary file 13 [file Image5.TIF]
