# Supplementary material for: Identification and Characterization of Genes Related to the Prognosis of Hepatocellular Carcinoma Based on Single-Cell Sequencing
Source: Pathol Oncol Res. 2022 Aug 25;28:1610199. doi: 10.3389/pore.2022.1610199 (PMC9454301; doi:10.3389/pore.2022.1610199)
Supplement: Supplementary file 2 [file Table1.DOCX]

Table S1. The R code used for the data analyses.

source('Z://projects/codes/mg_base.R')

setwd('E:/wtl/project/202006/HCC')

display_color<-function(colors=mg_colors,

start=1,

end =10) {

pie(rep(1, length(colors[start:end])),

col = colors[start:end],

border = colors[start:end],

labels = colors[start:end],

main = "Colors")

}

display_color(colors = mg_colors,

start = 1,

end = 15)

display_color()

dev.off()

library(RColorBrewer)

display.brewer.all(type = "seq")

display.brewer.all(type = "qual")

display.brewer.all(type = "div")

color12 <- brewer.pal(12, 'Paired')

color9 <- brewer.pal(8, 'Set2')

color9_1 <- brewer.pal(8, 'Set1')

display_color(colors = color9,

start = 1,

end = 9)

#2038个基因16880个细胞的表达谱

load('Z:/users/wangtl_248/202006/hcl_cell_types.RData')

scRNA_cell_type

#预测的细胞类型

hcl_cell=read.table('Z:/users/wangtl_248/202006/hcl_cell_types.txt',sep='\t',header = T)

#20个clust

scRNA_clust=read.table('Z:/users/wangtl_248/202006/monocle/monocle.clust.txt',sep='\t',header = T)

head(scRNA_clust)

scRNA_clust_tongji<-merge(data.frame(samples=scRNA_clust$sample,

cluster=scRNA_clust$Cluster,

patient=scRNA_clust$patient),

hcl_cell,by='samples')

cell_tongji=data.frame(table(scRNA_clust_tongji$cell))

colnames(cell_tongji)=c('cells','number')

cell_tongji=cell_tongji[order(cell_tongji$number),]

write.table(cell_tongji,'result/04cell_tongji/cell_tongji.txt',sep='\t',quote=F)

clust.cell_tongji<-data.frame(table(scRNA_clust_tongji$cluster,scRNA_clust_tongji$cell))

head(clust.cell_tongji)

colnames(clust.cell_tongji)=c('Cluster','cells','number')

library(reshape)

clust.cell_tongji1 <- cast(clust.cell_tongji,cells~Cluster)

dim(clust.cell_tongji1)

#write.table(clust.cell_tongji1,'')

#细胞亚群中病人来源的统计

scRNA_clust_tongji2=table(scRNA_clust_tongji$cluster,scRNA_clust_tongji$patient)

write.table(scRNA_clust_tongji2,'result/04cell_tongji/cell_patient.tongji.txt',sep='\t',quote=F)

library(ggsci)

colors=c(pal_npg("nrc", alpha = 0.8)(10) )

scRNA_clust_tongji2=crbind2DataFrame(scRNA_clust_tongji2)

scRNA_clust_tongji3=t(data.frame(scRNA_clust_tongji2))

plotMutiBar(scRNA_clust_tongji3[,1:10])

plotMutiBar(scRNA_clust_tongji3[,11:20])

pdf('result/04cell_tongji/S1.pdf',he=15,wi=15)

ggpubr::ggarrange(plotMutiBar(scRNA_clust_tongji3[,1:10],

showValue = T,showLine = F)

,plotMutiBar(scRNA_clust_tongji3[,11:20],

showValue = T,showLine = F)

,ncol = 2,nrow = 1,

labels = toupper(letters)[1:2],align = "hv")

dev.off()

#先剔除为0的cell

dim(clust.cell_tongji)

clust.cell_tongji=clust.cell_tongji[clust.cell_tongji$number>0,]

write.table(clust.cell_tongji,'result/04cell_tongji/clust.cell_tongji.txt',sep='\t',quote=F)

#clust1中细胞的组成

clust1.cell_tongji1=clust.cell_tongji[clust.cell_tongji$Cluster==1,]

clust1.cell_tongji2=clust1.cell_tongji1[which(clust1.cell_tongji1$number>0),]

chisq.test(clust1.cell_tongji2[,3])

#clust2中细胞的组成

clust2.cell_tongji1=clust.cell_tongji[clust.cell_tongji$Cluster==2,]

clust2.cell_tongji2=clust2.cell_tongji1[which(clust2.cell_tongji1$number>0),]

chisq.test(clust2.cell_tongji2[,3])

#clust3中细胞的组成

clust3.cell_tongji1=clust.cell_tongji[clust.cell_tongji$Cluster==3,]

clust3.cell_tongji2=clust3.cell_tongji1[which(clust3.cell_tongji1$number>0),]

chisq.test(clust3.cell_tongji2[,3])

#clust4中细胞的组成

clust4.cell_tongji1=clust.cell_tongji[clust.cell_tongji$Cluster==4,]

clust4.cell_tongji1=clust4.cell_tongji1[which(clust4.cell_tongji1$number>0),]

chisq.test(clust4.cell_tongji1[,3])

#clust5中细胞的组成

clust5.cell_tongji1=clust.cell_tongji[clust.cell_tongji$Cluster==5,]

clust5.cell_tongji1=clust5.cell_tongji1[which(clust5.cell_tongji1$number>0),]

chisq.test(clust5.cell_tongji1[,3])

#clust6中细胞的组成

clust6.cell_tongji1=clust.cell_tongji[clust.cell_tongji$Cluster==6,]

clust6.cell_tongji1=clust6.cell_tongji1[which(clust6.cell_tongji1$number>0),]

chisq.test(clust6.cell_tongji1[,3])

#clust7中细胞的组成

clust7.cell_tongji1=clust.cell_tongji[clust.cell_tongji$Cluster==7,]

clust7.cell_tongji1=clust7.cell_tongji1[which(clust7.cell_tongji1$number>0),]

chisq.test(clust7.cell_tongji1[,3])

#clust8中细胞的组成

clust8.cell_tongji1=clust.cell_tongji[clust.cell_tongji$Cluster==8,]

clust8.cell_tongji1=clust8.cell_tongji1[which(clust8.cell_tongji1$number>0),]

chisq.test(clust8.cell_tongji1[,3])

#clust9中细胞的组成

clust9.cell_tongji1=clust.cell_tongji[clust.cell_tongji$Cluster==9,]

clust9.cell_tongji1=clust9.cell_tongji1[which(clust9.cell_tongji1$number>0),]

chisq.test(clust9.cell_tongji1[,3])

#clust10中细胞的组成

clust10.cell_tongji1=clust.cell_tongji[clust.cell_tongji$Cluster==10,]

clust10.cell_tongji1=clust10.cell_tongji1[which(clust10.cell_tongji1$number>0),]

chisq.test(clust10.cell_tongji1[,3])

#clust11中细胞的组成

clust11.cell_tongji1=clust.cell_tongji[clust.cell_tongji$Cluster==11,]

clust11.cell_tongji1=clust11.cell_tongji1[which(clust11.cell_tongji1$number>0),]

chisq.test(clust11.cell_tongji1[,3])

#clust12中细胞的组成

clust12.cell_tongji1=clust.cell_tongji[clust.cell_tongji$Cluster==12,]

clust12.cell_tongji1=clust12.cell_tongji1[which(clust12.cell_tongji1$number>0),]

chisq.test(clust12.cell_tongji1[,3])

#clust13中细胞的组成

clust13.cell_tongji1=clust.cell_tongji[clust.cell_tongji$Cluster==13,]

clust13.cell_tongji1=clust13.cell_tongji1[which(clust13.cell_tongji1$number>0),]

chisq.test(clust13.cell_tongji1[,3])

#clust14中细胞的组成

clust14.cell_tongji1=clust.cell_tongji[clust.cell_tongji$Cluster==14,]

clust14.cell_tongji1=clust14.cell_tongji1[which(clust14.cell_tongji1$number>0),]

chisq.test(clust14.cell_tongji1[,3])

#clust15中细胞的组成

clust15.cell_tongji1=clust.cell_tongji[clust.cell_tongji$Cluster==15,]

clust15.cell_tongji1=clust15.cell_tongji1[which(clust15.cell_tongji1$number>0),]

chisq.test(clust15.cell_tongji1[,3])

#clust16中细胞的组成

clust16.cell_tongji1=clust.cell_tongji[clust.cell_tongji$Cluster==16,]

clust16.cell_tongji1=clust16.cell_tongji1[which(clust16.cell_tongji1$number>0),]

chisq.test(clust16.cell_tongji1[,3])

#clust17中细胞的组成

clust17.cell_tongji1=clust.cell_tongji[clust.cell_tongji$Cluster==17,]

clust17.cell_tongji1=clust17.cell_tongji1[which(clust17.cell_tongji1$number>0),]

chisq.test(clust17.cell_tongji1[,3])

#clust18中细胞的组成

clust18.cell_tongji1=clust.cell_tongji[clust.cell_tongji$Cluster==18,]

clust18.cell_tongji1=clust18.cell_tongji1[which(clust18.cell_tongji1$number>0),]

chisq.test(clust18.cell_tongji1[,3])

#clust19中细胞的组成

clust19.cell_tongji1=clust.cell_tongji[clust.cell_tongji$Cluster==19,]

clust19.cell_tongji1=clust19.cell_tongji1[which(clust19.cell_tongji1$number>0),]

chisq.test(clust19.cell_tongji1[,3])

#clust20中细胞的组成

clust20.cell_tongji1=clust.cell_tongji[clust.cell_tongji$Cluster==20,]

clust20.cell_tongji1=clust20.cell_tongji1[which(clust20.cell_tongji1$number>0),]

chisq.test(clust20.cell_tongji1[,3])

#读取18个clust的特征基因

clust.uniq<-read.table('Z:/users/wangtl_248/202006/04.cor/clust.unique.gene.txt',sep='\t',header=T)

clust.marker<-read.table('Z:/users/wangtl_248/202006/scRNA/gene.marker.txt',sep='\t',header=T)

head(clust.uniq)

head(clust.marker)

tcga_exp<-read.table('Z:/TCGA/Matrix/mRNA_TPM_Symbol/Merge_TCGA-LIHC_TPM.txt',sep='\t',header = T,row.names = 1)

tcga_cli<-read.table('E:/wtl/project/PMC6066282-TCGA-CDR-clinical.txt',sep='\t',header = T,row.names = 1)

tcga_time<-data.frame(sample=rownames(tcga_cli),

OS=tcga_cli$OS,

OS.time=tcga_cli$OS.time,

DSS=tcga_cli$DSS,

DSS.time=tcga_cli$DSS.time,

DFI=tcga_cli$DFI,

DFI.time=tcga_cli$DFI.time,

PFI=tcga_cli$PFI,

PFI.time=tcga_cli$PFI.time)

head(tcga_time)

tcga_clis=read.table('E:/wtl/project/202006/HCC/TCGA-LIHC_Merge.txt',sep='\t',header=T,row.names=1)

tcga_cli<-merge(tcga_time,

data.frame(sample=rownames(tcga_clis),tcga_clis),

by='sample')

dim(tcga_cli)

tcga_cli$sample=gsub('-','\\.',tcga_cli$sample)

rownames(tcga_cli)=paste0(tcga_cli$sample,'.01')

head(tcga_cli)

com_sample=intersect(rownames(tcga_cli),colnames(tcga_exp))

tcga.exp=tcga_exp[,com_sample]

tcga_cli=tcga_cli[com_sample,]

dim(tcga.exp)

dim(tcga_cli)

library(pheatmap)

clust.marker

com_gene=intersect(clust.marker$x,rownames(tcga.exp))

tcga.exp1=tcga.exp[com_gene,]

tcga.exp2 <- tcga.exp1[which(apply(tcga.exp1,1,function(x){return(sum(x==0))})<ncol(tcga.exp1)),]

tcga.exp1=tcga.exp1[tcga.exp1>0]

bk=unique(c(seq(-1,1,length=100)))

clust.row=pheatmap(tcga.exp2,show_rownames = F,scale='row',

cutree_rows = 2,breaks = bk,clustering_method='ward.D')

clust.col=pheatmap(tcga.exp2,show_rownames = F,scale='row',

cutree_cols = 2,breaks = bk,clustering_method='ward.D')

row_anno = data.frame(group = factor(paste0('group',

cutree(clust.row$tree_row,2))))

rownames(row_anno) = rownames(tcga.exp2)

col_anno = data.frame(clust = factor(paste0('cluster',

cutree(clust.col$tree_col,2))))

rownames(col_anno) = colnames(tcga.exp2)

anno_cols<-list(clust=c('cluster1'='#E64B35','cluster2'='#4DBBD5'))

write.table(tcga.exp2,'result/tcga.1930.gene.exp.txt',sep='\t',quote = F)

pdf('result/05subtype2/tcga_subtype_2.pheatmap.pdf',wi=7,he=7)

pheatmap(tcga.exp2,scale='row',

breaks=bk,clustering_method='ward.D',

annotation_col=col_anno,annotation_colors = anno_cols,

annotation_row=row_anno,

cluster_cols=T,cluster_rows=T,

show_rownames=F,show_colnames=F,

color=colorRampPalette(c("navy","white","firebrick3"))(100))

dev.off()

row_anno1=data.frame(gene=rownames(row_anno),group=row_anno$group)

write.table(row_anno1,'result/group.txt',sep='\t',quote = F,row.names = F)

#生存分析

tcga_subtype2<-merge(data.frame(sample=rownames(col_anno),

col_anno),

data.frame(sample=paste0(gsub('-','\\.',tcga_time$sample),'.01'),

tcga_time)

,by='sample')

tcga_subtype2_os<-data.frame(tcga_subtype2$OS.time/365,

tcga_subtype2$OS,

tcga_subtype2$clust)

rownames(tcga_subtype2_os)=tcga_subtype2$sample

tcga_subtype2_os=na.omit(tcga_subtype2_os)

write.table(tcga_subtype2_os,'result/subtype.os.txt',sep='\t',quote = F)

pdf('result/05subtype2/tcga_subtype_2.km.os.pdf',wi=7,he=7)

ggplotKMCox(tcga_subtype2_os,labs = c('cluster1','cluster2'))

dev.off()

#GSVA分析####

dim(tcga.exp)

library(GSVA)

library(limma)

library(GSEABase)

rt1<-tcga.exp

exp1=as.matrix(log2(rt1+1))

dimnames=list(rownames(exp1),colnames(exp1))

mat1=matrix(as.numeric(as.matrix(exp1)),nrow=nrow(exp1),dimnames=dimnames)

mat1=avereps(mat1)

mat1=normalizeBetweenArrays(mat1)

gmtFile='Z:/projects/codes/source/c2.cp.kegg.v7.0.symbols.gmt'

c3gsc2=getGmt( gmtFile,

collectionType=BroadCollection(category="c3"),

geneIdType=SymbolIdentifier())

gsvaOut1=gsva(mat1,

c3gsc2,

min.sz=10,

max.sz=500,

verbose=TRUE,

parallel.sz=1)

rownames(gsvaOut1)=gsub('KEGG_','',rownames(gsvaOut1))

write.table(gsvaOut1,file="result/GSVA.txt",sep="\t",quote=F)

group_subtype=data.frame(sample=rownames(col_anno),

clust=col_anno)

rownames(group_subtype)=rownames(col_anno)

subtype.kegg_deg=mg_limma_DEG(gsvaOut1,group_subtype$clust,'cluster1','cluster2')

subtype.kegg=subtype.kegg_deg$DEG[abs(subtype.kegg_deg$DEG$t)>6,]

dim(subtype.kegg)

write.table(group_subtype,'result/subtype.txt',sep='\t',row.names = F)

group_subtype=group_subtype[order(group_subtype$clust,decreasing=T),]

bk1=unique(c(seq(-0.5,0.5,length=100)))

pdf('result/05subtype2/gsva.kegg.pdf',he=8,wi=15)

pheatmap(gsvaOut1[rownames(subtype.kegg),rownames(group_subtype)],scale='none',

breaks=bk1,clustering_method='ward.D',annotation_colors = anno_cols,

annotation_col=col_anno,

#annotation_row=row_anno,

cluster_cols=F,cluster_rows=T,

show_rownames=T,show_colnames=F,

color=colorRampPalette(c("navy","white","firebrick3"))(100))

dev.off()

#TME####

#使用CIBERSORT方法计算22中免疫评分

tcga_immnue.cell=immu_CIBERSORT(exp_data=tcga.exp)

tcga_immnue.cell1=tcga_immnue.cell[,-c(25,24,23)]

tcga_immnue.cell2=merge(data.frame(sample=rownames(col_anno),

col_anno),

data.frame(sample=rownames(tcga_immnue.cell1),

tcga_immnue.cell1),by='sample')

pdf('result/05subtype2/immnu.cell.pdf',he=8,wi=15)

groupViolin(data = tcga_immnue.cell2[,-c(1,2)],

group = tcga_immnue.cell2$clust,

ylab='Immune Infiltration',ylim=c(0,0.4),

group_col = c('cluster1'='#E64B35','cluster2'='#4DBBD5'))

dev.off()

#estimate包计算样本的免疫评分函数

tcga_immue=immu_estimate(tcga.exp,platform='illumina',isTCGA=T)

tcga_immue1=tcga_immue

rownames(tcga_immue1)=gsub('-','\\.',rownames(tcga_immue1))

tcga_immue1_subtype=merge(data.frame(sample=rownames(tcga_immue1),

tcga_immue1),

group_subtype,by='sample')

pdf('result/05subtype2/estimate1.pdf',he=7,wi=7)

groupViolin(data = tcga_immue1_subtype[,-c(1,5)],group = tcga_immue1_subtype$clust,ylab='Immune Infiltration',group_col = c('cluster1'='#E64B35','cluster2'='#4DBBD5'))

dev.off()

#PD-1（PDCD1：程序性细胞死亡蛋白1）和CTLA-4（细胞毒性T淋巴细胞相关蛋白4）在两个亚型中表达

rownames(tcga.exp)

immgene=t(tcga.exp[c('PDCD1','CTLA4'),])

immgene.subtype=merge(group_subtype,

data.frame(sample=rownames(immgene),

immgene),by='sample')

pdf('result/05subtype2/PDCD1.CTLA4.subtype.pdf',wi=7,he=7)

groupViolin(log2(immgene.subtype[,c(3,4)]+1),immgene.subtype$clust,ylab='log2exp',group_col = c('cluster1'='#E64B35','cluster2'='#4DBBD5'))

dev.off()

#两个clust在6个免疫亚型中分布####

imm.subtype=read.csv('E:/wtl/public/TCGA_immune_subtype_mmc2.csv',sep=',',header=T,row.names = 1)

rownames(imm.subtype)=paste0(gsub('-','\\.',rownames(imm.subtype)),'.01')

imm.subtype1=imm.subtype[intersect(group_subtype$sample,rownames(imm.subtype)),]

imm.subtype2=merge(group_subtype,

data.frame(sample=rownames(imm.subtype1),

subtype=imm.subtype1$Immune.Subtype),

by='sample')

head(imm.subtype2)

imm.subtype3=as.data.frame(table(imm.subtype2$clust,imm.subtype2$subtype))

colnames(imm.subtype3)=c('marker-clust','immnue.clust','number')

imm.subtype3.clus1=imm.subtype3[imm.subtype3$`marker-clust`=='cluster1',]

imm.subtype3.clus2=imm.subtype3[imm.subtype3$`marker-clust`=='cluster2',]

imm.subtype3.all=as.data.frame(table(imm.subtype2$subtype))

#table(imm.subtype2$subtype)

pie(x = imm.subtype3.all$Freq,

labels = c('c1','c2','c3','c4','C6'),

col=rainbow(5))

piepercent<- paste(round(100*imm.subtype3.all$Freq/sum(imm.subtype3.all$Freq), 2), "%")

pdf('result/05subtype2/tcga.immnue.tongji.pdf',he=5,wi=5)

pie(x = imm.subtype3.all$Freq,

labels = piepercent,

col=rainbow(5))

dev.off()

piepercent1<- paste(round(100*imm.subtype3.clus1$number/sum(imm.subtype3.clus1$number), 2), "%")

pdf('result/05subtype2/tcga.immnue.subtype.tongji1.pdf',he=5,wi=5)

pie(x = imm.subtype3.clus1$number,

labels = piepercent1,

col=rainbow(4))

dev.off()

pie(x = imm.subtype3.clus1$number,

labels = c('c1','c2','c3','c4'),

col=rainbow(4))

piepercent2<- paste(round(100*imm.subtype3.clus2$number/sum(imm.subtype3.clus2$number), 2), "%")

pdf('result/05subtype2/tcga.immnue.subtype.tongji2.pdf',he=5,wi=5)

pie(x = imm.subtype3.clus2$number,

labels = piepercent2,

col=rainbow(5))

dev.off()

pie(x = imm.subtype3.clus2$number,

labels = c('c1','c2','c3','c4','c6'),

col=rainbow(5))

#km曲线

imm.subtype.km<-merge(imm.subtype2,

data.frame(sample=paste0(gsub('-','\\.',tcga_time$sample),'.01'),

tcga_time)

,by='sample')

head(imm.subtype.km)

imm.subtype.os=data.frame(imm.subtype.km$OS.time/365,

imm.subtype.km$OS,

imm.subtype.km$subtype)

imm.subtype.os=na.omit(imm.subtype.os)

pdf('result/05subtype2/tcga.immnue.subtype.km.os.pdf',he=7,wi=7)

ggplotKMCox(imm.subtype.os,labs=c('C1','C2','C3','C4','C6'))

dev.off()

#去掉C6亚型

imm.subtype.os1=imm.subtype.os[imm.subtype.os$imm.subtype.km.subtype!='c6',]

ggplotKMCox(imm.subtype.os1)

save.image('0615.RData')

#snv和cnv突变分析

library('maftools')

library(data.table)

tcga_maf<- read.maf(maf='z:/TCGA/Matrix/mutect2/TCGA.LIHC.mutect.a630f0a0-39b3-4aab-8181-89c1dde8d3e2.DR-10.0.somatic.maf.gz')

write.mafSummary(maf=tcga_maf, basename="TCGA")

#查看前15个基因

oncoplot(maf = tcga_maf,

top = 15,

bgCol = "#FFFFFF")

oncoplot(maf = tcga_maf,

genes = c('CYP1A1','TP53','NQO1','ALDH2','EPHX1'),

# top = 15,

bgCol = "#FFFFFF")

tcga_mutant_info <- read.delim('TCGA_maftools.maf', header = T, stringsAsFactors = F)

table(tcga_mutant_info$Variant_Classification)

tcga_mutant_info1 <- tcga_mutant_info[, c("Hugo_Symbol",

"Variant_Classification",

"Tumor_Sample_Barcode")]

colnames(tcga_mutant_info1) <- c("Symbol", "Variant", "Sample")

table(tcga_mutant_info1$Variant)

tcga_mutant_info1$Variant[tcga_mutant_info1$Variant == "3'Flank"] <- 'Other'

tcga_mutant_info1$Variant[tcga_mutant_info1$Variant == "3'UTR"] <- 'Other'

tcga_mutant_info1$Variant[tcga_mutant_info1$Variant == "5'Flank"] <- 'Other'

tcga_mutant_info1$Variant[tcga_mutant_info1$Variant == "5'UTR"] <- 'Other'

tcga_mutant_info1$Variant[tcga_mutant_info1$Variant == "IGR"] <- 'Other'

tcga_mutant_info1$Variant[tcga_mutant_info1$Variant == "Intron"] <- 'Other'

tcga_mutant_info1$Variant[tcga_mutant_info1$Variant == "Nonstop_Mutation"] <- 'Other'

tcga_mutant_info1$Variant[tcga_mutant_info1$Variant == "RNA"] <- 'Other'

tcga_mutant_info1$Variant[tcga_mutant_info1$Variant == "Silent"] <- 'Other'

tcga_mutant_info1$Variant[tcga_mutant_info1$Variant == "Splice_Region"] <- 'Other'

tcga_mutant_info1$Variant[tcga_mutant_info1$Variant == "Translation_Start_Site"] <- 'Other'

tcga_mutant_info1$Variant[tcga_mutant_info1$Variant == "Splice_Site"] <- 'Other'

tcga_mutant_info1$Variant[tcga_mutant_info1$Variant == "Frame_Shift_Del"] <- 'Frame Shift InDel'

tcga_mutant_info1$Variant[tcga_mutant_info1$Variant == "Frame_Shift_Ins"] <- 'Frame Shift InDel'

tcga_mutant_info1$Variant[tcga_mutant_info1$Variant == "In_Frame_Del"] <- 'In Frame InDel'

tcga_mutant_info1$Variant[tcga_mutant_info1$Variant == "In_Frame_Ins"] <- 'In Frame InDel'

tcga_mutant_info1$Variant[tcga_mutant_info1$Variant == "Missense_Mutation"] <- 'Missense'

tcga_mutant_info1$Variant[tcga_mutant_info1$Variant == "Nonsense_Mutation"] <- 'Nonsense'

tcga_mutant_info1 <- tcga_mutant_info1[!duplicated(tcga_mutant_info1[, c(1, 3)]), ]

write.csv(tcga_mutant_info1, file = 'result/09.snv.cnv/tcga_mutant_info1.csv', row.names = F)

head(tcga_mutant_info1)

library(tidyr)

library(dplyr)

tcga_mutant_info1=tcga_mutant_info1[,c(1,3,2)]

colnames(tcga_mutant_info1)=c('gene','sample','variant')

tcga_mutant_info1=unique(tcga_mutant_info1)

tcga_mutant_info2=tcga_mutant_info1 %>% group_by(variant) %>% mutate(id=1:n()) %>% ungroup() %>% spread(sample,variant) %>%select(-id)

colnames(tcga_mutant_info2) <- substr(colnames(tcga_mutant_info2), 1, 15)

tcga_mutant_info2=data.frame(tcga_mutant_info2)

tcga_mutant_info2[is.na(tcga_mutant_info2)] <- 'Wildtype'

tcga_mutant_info2=tcga_mutant_info2[!duplicated(tcga_mutant_info2$gene), ]

rownames(tcga_mutant_info2)=tcga_mutant_info2$gene

tcga_mutant_info2=tcga_mutant_info2[,-1]

tcga_CNV_info2 <- read.delim('data/all_thresholded.by_genes.txt',

header = T, row.names = 1, sep='\t',

stringsAsFactors = F, check.names = F)

tcga_CNV_info2 <- tcga_CNV_info2[, -c(1,2)]

max(tcga_CNV_info2)

min(tcga_CNV_info2)

colnames(tcga_CNV_info2)=gsub('-','\\.',colnames(tcga_CNV_info2))

cnv_com_samples <- intersect(colnames(tcga_CNV_info2),

tcga_subtype2$sample)

tcga_CNV_info2 <- tcga_CNV_info2[, cnv_com_samples]

tcga_CNV_info2[tcga_CNV_info2 == -1] <- 'Loss'

tcga_CNV_info2[tcga_CNV_info2 == -2] <- 'Loss'

tcga_CNV_info2[tcga_CNV_info2 == 1] <- 'Gain'

tcga_CNV_info2[tcga_CNV_info2 == 2] <- 'Gain'

tcga_CNV_info2[tcga_CNV_info2 == 0] <- 'Neutral'

tcga_mut_genes <- c('CYP1A1','TP53','NQO1','ALDH2','EPHX1')

length(tcga_mut_genes)

intersect(rownames(tcga_CNV_info2), tcga_mut_genes)

oncoplot(maf = tcga_maf,

# genes = tcga_mut_genes,

top = 15,

bgCol = "#FFFFFF")

tcga_CNV_info3 <- t(tcga_CNV_info2[tcga_mut_genes, ])

colnames(tcga_CNV_info3) <- paste0(colnames(tcga_CNV_info3), '_CNV')

tcga_CNV_info3 <- crbind2DataFrame(tcga_CNV_info3)

tcga_CNV_info3$samples <- rownames(tcga_CNV_info3)

intersect(rownames(tcga_mutant_info2), tcga_mut_genes)

tcga_mutant_info3 <- t(tcga_mutant_info2[tcga_mut_genes, ])

colnames(tcga_mutant_info3) <- paste0(colnames(tcga_mutant_info3), '_Mutant')

tcga_mutant_info3 <- crbind2DataFrame(tcga_mutant_info3)

tcga_mutant_info3$samples <- rownames(tcga_mutant_info3)

tcga_cnv_mut_cli <- merge(data.frame(samples=tcga_subtype2$sample,

cluster=tcga_subtype2$clust),

tcga_mutant_info3,

by= 'samples', all = T)

tcga_cnv_mut_cli <- merge(tcga_cnv_mut_cli, tcga_CNV_info3,

by = 'samples', all = T)

tcga_cnv_mut_cli <- tcga_cnv_mut_cli[!is.na(tcga_cnv_mut_cli$cluster), ]

tcga_cnv_mut_cli[is.na(tcga_cnv_mut_cli)] <- 'ZNA'

table(tcga_cnv_mut_cli$TP53_Mutant)

table(tcga_cnv_mut_cli$TP53_CNV)

# 绘制突变和拷贝数的分布图 ####################################

library(dplyr)

c('CYP1A1','TP53','NQO1','ALDH2','EPHX1')

# CYP1A1 ###########

color_CYP1A1_mut <- c(color12[1:5], "gray80","white")

names(color_CYP1A1_mut) <- c('Frame Shift InDel', 'In Frame InDel','Missense',

'Nonsense','Other','Wildtype', 'ZNA' )

color_CYP1A1_cnv <- c(color9_1[1:2], "gray80","white")

names(color_CYP1A1_cnv) <- c('Gain','Loss','Neutral', 'ZNA' )

# TP53 ###########

color_TP53_mut <- c(color12[1:5], "gray80","white")

names(color_TP53_mut) <- c('Frame Shift InDel', 'In Frame InDel','Missense',

'Nonsense','Other','Wildtype', 'ZNA' )

color_TP53_cnv <- c(color9_1[1:2], "gray80","white")

names(color_TP53_cnv) <- c('Gain','Loss','Neutral', 'ZNA' )

# NQO1 ###########

color_NQO1_mut <- c(color12[1:5], "gray80","white")

names(color_NQO1_mut) <- c('Frame Shift InDel', 'In Frame InDel','Missense',

'Nonsense','Other','Wildtype', 'ZNA' )

color_NQO1_cnv <- c(color9_1[1:2], "gray80","white")

names(color_NQO1_cnv) <- c('Gain','Loss','Neutral', 'ZNA' )

# ALDH2 ###########

color_ALDH2_mut <- c(color12[1:5], "gray80","white")

names(color_ALDH2_mut) <- c('Frame Shift InDel', 'In Frame InDel','Missense',

'Nonsense','Other','Wildtype', 'ZNA' )

color_ALDH2_cnv <- c(color9_1[1:2], "gray80","white")

names(color_ALDH2_cnv) <- c('Gain','Loss','Neutral', 'ZNA' )

# EPHX1 ###########

color_EPHX1_mut <- c(color12[1:5], "gray80","white")

names(color_EPHX1_mut) <- c('Frame Shift InDel', 'In Frame InDel','Missense',

'Nonsense','Other','Wildtype', 'ZNA' )

color_EPHX1_cnv <- c(color9_1[1:2], "gray80","white")

names(color_EPHX1_cnv) <- c('Gain','Loss','Neutral', 'ZNA' )

color_type=c('#E64B35', '#4DBBD5', '#00A087', '#3C5488')

names(color_type)=c('Cholesterol','Glycolysis','Mixed','Quiescent' )

library(ComplexHeatmap)

tcga_cnv_mut_cli <- arrange(tcga_cnv_mut_cli, cluster, CYP1A1_Mutant, TP53_Mutant,NQO1_Mutant, ALDH2_Mutant, EPHX1_Mutant)

ha = HeatmapAnnotation(df=tcga_cnv_mut_cli[, c("cluster", "CYP1A1_Mutant", "CYP1A1_CNV","TP53_Mutant", "TP53_CNV","NQO1_Mutant", "NQO1_CNV","ALDH2_Mutant", "ALDH2_CNV","EPHX1_Mutant", "EPHX1_CNV")],

col=list("type"=color_type,

"CYP1A1_Mutant"= color_CYP1A1_mut,"CYP1A1_CNV"=color_CYP1A1_cnv,

"TP53_Mutant"=color_TP53_mut,'TP53_CNV'=color_TP53_cnv,

'NQO1_Mutant'=color_NQO1_mut,'NQO1_CNV'=color_NQO1_cnv,

'ALDH2_Mutant'=color_ALDH2_mut,'ALDH2_CNV'=color_ALDH2_cnv,

'EPHX1_Mutant'=color_EPHX1_mut,'EPHX1_CNV'=color_EPHX1_cnv),

show_annotation_name = TRUE,

gap=unit(1, "mm"), na_col="grey")

library(circlize)

heat_colors2 <- colorRamp2(c(-2, 0, 2), c("midnightblue", "white", "red"))

Heatmap(tcga.exp[c('TP53', 'CTNNB1'), tcga_cnv_mut_cli$samples],

name="Expression Score",

top_annotation = ha,

cluster_rows = F,

border = 'grey',

col=heat_colors2,

color_space = "RGB",

cluster_columns = F,

show_column_dend=T,

row_order=NULL,

column_order=NULL,

show_column_names = F,

show_row_names = T,

row_names_gp = gpar(fontsize = 5),

gap = unit(1, "mm"),

column_title = "",

column_title_gp = gpar(fontsize = 5),

width=unit(30, "cm"),

show_heatmap_legend = TRUE,

heatmap_legend_param=list(labels_gp = gpar(fontsize = 6),

title_gp = gpar(fontsize = 6, fontface = "bold")))

dev.off()

#背景校正后

tcga_immue1_subtype1=data.frame(

StromalScore=tcga_immue1_subtype$StromalScore/tcga_immue1_subtype$ESTIMATEScore,

ImmuneScore=tcga_immue1_subtype$ImmuneScore/tcga_immue1_subtype$ESTIMATEScore,

clust=tcga_immue1_subtype$clust)

pdf('result/05subtype2/estimate1.pdf',he=7,wi=7)

groupViolin(data = tcga_immue1_subtype1[,-3],group = tcga_immue1_subtype1$clust,xangle = 0,ylab='Immune Infiltration')

dev.off()

#两个亚型的样本分组

col_anno1=data.frame(sample=rownames(col_anno),clust=col_anno$clust)

rownames(col_anno1)=col_anno1$sample

clust1_sample=rownames(col_anno1[which(col_anno1$clust=='cluster1'),])

row_anno1=data.frame(gene=rownames(row_anno),group=row_anno$group)

rownames(row_anno1)=row_anno1$gene

group1_gene<-rownames(row_anno1[row_anno1$group=='group1',])

group2_gene<-rownames(row_anno1[row_anno1$group=='group2',])

#富集分析

group1_gene_res=enrichmentORA(group1_gene,

mp_dbs=c('pathway_KEGG',

'geneontology_Biological_Process',

'geneontology_Cellular_Component',

'geneontology_Molecular_Function'))

pdf('result/05subtype2/enrich.group1.pdf',wi=15,he=9)

dotplot_batch(group1_gene_res,top = 10)

dev.off()

group2_gene_res=enrichmentORA(group2_gene,

mp_dbs=c('pathway_KEGG',

'geneontology_Biological_Process',

'geneontology_Cellular_Component',

'geneontology_Molecular_Function'))

pdf('result/05subtype2/enrich.group2.pdf',wi=15,he=9)

dotplot_batch(group2_gene_res,top = 10)

dev.off()

write.table(group1_gene_res,'result/05subtype2/enrich.group1.txt',sep='\t',quote = F)

write.table(group2_gene_res,'result/05subtype2/enrich.group2.txt',sep='\t',quote = F)

#差异分析####

tcga_exp.guolv.deg<-mg_limma_DEG(log2(tcga_exp.guolv[,rownames(col_anno)]+1),col_anno$clust,'cluster1','cluster2')

pdf('result/05subtype2/cluster1vscluster2.volcano.pdf',he=7,wi=7)

mg_volcano(logfc =tcga_exp.guolv.deg$DEG$logFC,

pvalue = tcga_exp.guolv.deg$DEG$adj.P.Val,

cutFC = 1,cutPvalue = 0.05)

dev.off()

write.table(tcga_exp.guolv.deg$DEG,'result/07.deg.gene/cluster1vscluster2.all.gene.txt',sep='\t',quote=F)

tcga_exp.guolv.deg.gene<-tcga_exp.guolv.deg$DEG[abs(tcga_exp.guolv.deg$DEG$logFC)>1 &tcga_exp.guolv.deg$DEG$adj.P.Val<0.05,]

write.table(tcga_exp.guolv.deg.gene,'result/07.deg.gene/cluster1vscluster2.deg.gene.txt',sep='\t',quote=F)

dim(tcga_exp.guolv.deg.gene[tcga_exp.guolv.deg.gene$logFC>0,])

dim(tcga_exp.guolv.deg.gene[tcga_exp.guolv.deg.gene$logFC<0,])

dim(tcga_exp.guolv.deg.gene)

#富集分析

#表达上调的基因

up_gene=rownames(tcga_exp.guolv.deg.gene[tcga_exp.guolv.deg.gene$logFC>0,])

#up_gene_res=enrichmentORA(up_gene,mp_dbs=c('pathway_KEGG',

# 'geneontology_Biological_Process',

# 'geneontology_Cellular_Component',

# 'geneontology_Molecular_Function'))

up_gene_res.kegg=up_gene_res[up_gene_res$DB=='pathway_KEGG',]

write.table(up_gene_res.kegg,'result/07.deg.gene/up.kegg.txt',sep='\t',quote=F)

pdf('result/07deg_subtype/enrich.up.pdf',wi=15,he=9)

dotplot_batch(up_gene_res,top = 10)

dev.off()

#表达下调的基因

down_gene=rownames(tcga_exp.guolv.deg.gene[tcga_exp.guolv.deg.gene$logFC<0,])

#down_gene_res=enrichmentORA(down_gene,mp_dbs=c('pathway_KEGG',

# 'geneontology_Biological_Process',

# 'geneontology_Cellular_Component',

# 'geneontology_Molecular_Function'))

pdf('result/07deg_subtype/enrich.down.pdf',wi=15,he=9)

dotplot_batch(down_gene_res,top = 10)

dev.off()

down_gene_res.kegg<-down_gene_res[down_gene_res$DB=='pathway_KEGG',]

write.table(down_gene_res.kegg,'result/07.deg.gene/down.kegg.txt',sep='\t',quote=F)

#kegg通路最显著的前10个

up.kegg<-read.table('result/05subtype2/up.kegg.txt',sep='\t',header = T)

bubble1=ggplot(data = up.kegg,

aes(x = enrichmentRatio, y = description)) +

xlab('Enrichment Ratio')+

geom_point(aes(size = size,color = -log10(FDR))) +

scale_color_gradient(low = ggsci::pal_npg()(2)[1],

high = ggsci::pal_npg()(2)[2])+

ggsci::scale_fill_npg()+

ggplot2::theme_bw()+

theme(axis.title.y=element_blank(),

axis.text.y=element_text(family="Times",face="plain"),

strip.text.y = element_text(family="Times",face="plain",hjust = 0.5,angle = 0),

axis.text.x=element_text(family="Times",face="plain"),

plot.title = element_text(hjust = 0.5,family="Times",face="plain") ,

axis.title.x=element_text(family="Times",face="plain"),

legend.title = element_text(family="Times",face="plain"),

legend.text = element_text(family="Times",face="plain"))+

facet_grid(class~.,scales = "free", space = "free")

down.kegg<-read.table('result/05subtype2/down.kegg.txt',sep='\t',header = T)

bubble2=ggplot(data = down.kegg,

aes(x = enrichmentRatio, y = description)) +

xlab('Enrichment Ratio')+

geom_point(aes(size = size,color = -log10(FDR))) +

scale_color_gradient(low = ggsci::pal_npg()(2)[1],

high = ggsci::pal_npg()(2)[2])+

ggsci::scale_fill_npg()+

ggplot2::theme_bw()+

theme(axis.title.y=element_blank(),

axis.text.y=element_text(family="Times",face="plain"),

strip.text.y = element_text(family="Times",face="plain",hjust = 0.5,angle = 0),

axis.text.x=element_text(family="Times",face="plain"),

plot.title = element_text(hjust = 0.5,family="Times",face="plain") ,

axis.title.x=element_text(family="Times",face="plain"),

legend.title = element_text(family="Times",face="plain"),

legend.text = element_text(family="Times",face="plain"))+

facet_grid(class~.,scales = "free", space = "free")

pdf('result/05subtype2/enrich.pdf',he=15,wi=25)

ggpubr::ggarrange(bubble1,bubble2,

ncol = 2,nrow = 1,

labels = toupper(letters)[1:2],align = "hv")

dev.off()

#两个亚型的GSEA分析

write.table(log2(tcga_exp.guolv[,rownames(col_anno)]+1),'result/GSEA/TCGA.exp.data.txt',sep='\t',quote=F)

write.table(col_anno,'result/GSEA/group.txt',sep='\t',quote=F)

tcga_exp.guolv.deg<-mg_limma_DEG(log2(tcga_exp.guolv[,rownames(col_anno)]+1),col_anno$clust,'cluster1','cluster2')

mg_RunGSEA(mod = 'exp_group',exp_Path = 'result/GSEA/TCGA.exp.data.txt'

,sample_group_path = 'result/GSEA/group.txt'

,outFolder = 'result/GSEA/'

,gmt_Path = 'KEGG',outLog=F)

tcga_GSEA=parseGSEAResult('result/GSEA/my_analysis.Gsea.1592364168099/')

plot_GSEA_By_node(tcga_GSEA,index=1,TermName=NULL,left='Negative',right='Positive')

inds1=which(tcga_GSEA$EnrichTable$NES>0 & tcga_GSEA$EnrichTable$NP<0.05)

inds2=which(tcga_GSEA$EnrichTable$NES<0 & tcga_GSEA$EnrichTable$NP<0.05)

tcga_GSEA$EnrichTable[inds1,]$Term

tcga_GSEA$EnrichTable[inds2,]$Term

pdf('result/GSEA/GSEA.result1.pdf',he=9,wi=12)

plot_GSEA_By_nodes(tcga_GSEA,indexs=inds1[1:10])

dev.off()

pdf('result/GSEA/GSEA.result2.pdf',he=9,wi=12)

plot_GSEA_By_nodes(tcga_GSEA,indexs=inds2[1:10])

dev.off()

write.table(tcga_GSEA$EnrichTable,'result/GSEA/GSEA.result.txt',sep='\t',quote=F)

dim(tcga_exp.guolv.deg.gene)

#临床特征的比较

group_subtype

tcga_cli

subtype.cli<-merge(group_subtype,

data.frame(sample=rownames(tcga_cli),

tcga_cli[,-1]),by='sample')

subtype.cli1=subtype.cli

subtype.cli1$OS=gsub(0,'alive',subtype.cli1$OS)

subtype.cli1$OS=gsub(1,'dead',subtype.cli1$OS)

#生存状态

statu=table(subtype.cli1$clust,subtype.cli1$OS)

plotMutiBar(statu,ist = T,legTitle = 'status',showValue = T)

#性别

gender=table(subtype.cli1$clust,subtype.cli1$A18_Sex)

plotMutiBar(gender,ist = T,legTitle = 'gender',showValue = T)

#T

subtype.cli1$A3_T=gsub('a','',subtype.cli1$A3_T)

subtype.cli1$A3_T=gsub('b','',subtype.cli1$A3_T)

T.fenqi=table(subtype.cli1$clust,subtype.cli1$A3_T)

T.fenqi1=T.fenqi[,-1]

plotMutiBar(T.fenqi1,ist = T,legTitle = 'T',showValue = T)

#N分期

N.fenqi=table(subtype.cli1$clust,subtype.cli1$A4_N)

N.fenqi1=N.fenqi[,-1]

plotMutiBar(N.fenqi1,ist = T,legTitle = 'N',showValue = T)

#M分期

M.fenqi=table(subtype.cli1$clust,subtype.cli1$A5_M)

plotMutiBar(M.fenqi,ist = T,legTitle = 'M',showValue = T)

#stage分期

subtype.cli1$A6_Stage=gsub('A','',subtype.cli1$A6_Stage)

subtype.cli1$A6_Stage=gsub('B','',subtype.cli1$A6_Stage)

subtype.cli1$A6_Stage=gsub('C','',subtype.cli1$A6_Stage)

stage=table(subtype.cli1$clust,subtype.cli1$A6_Stage)

stage1=stage[,-1]

plotMutiBar(stage1,ist = T,legTitle = 'stage',showValue = T)

#grade

grade=table(subtype.cli1$clust,subtype.cli1$A7_Grade)

grade1=grade[,-5]

plotMutiBar(grade1,ist = T,legTitle = 'grade',showValue = T)

#绘图

pdf('result/subtype.clitongji.pdf',he=15,wi=15)

ggpubr::ggarrange(plotMutiBar(gender,ist = T,legTitle = 'gender',showValue = T),

plotMutiBar(T.fenqi1,ist = T,legTitle = 'T',showValue = T),

plotMutiBar(N.fenqi1,ist = T,legTitle = 'N',showValue = T),

plotMutiBar(M.fenqi,ist = T,legTitle = 'M',showValue = T),

plotMutiBar(stage1,ist = T,legTitle = 'stage',showValue = T),

plotMutiBar(grade1,ist = T,legTitle = 'grade',showValue = T),

ncol = 3,nrow = 2,

labels = toupper(letters)[1:6],align = "hv")

dev.off()

#关键基因的筛选

rownames(tcga_exp.guolv.deg.gene)

tcga_exp.guolv.deg.gene1<-tcga_exp.guolv.deg.gene[

abs(tcga_exp.guolv.deg.gene$logFC)>2,]

dim(tcga_exp.guolv.deg.gene1[tcga_exp.guolv.deg.gene1$logFC>0,])

dim(tcga_exp.guolv.deg.gene1[tcga_exp.guolv.deg.gene1$logFC<0,])

dim(tcga_exp.guolv.deg.gene1)

write.table(rownames(tcga_exp.guolv.deg.gene1),'result/PPI/genes.txt',sep='\t',quote = F)

cytoscape_up_gene=rownames(tcga_exp.guolv.deg.gene1[tcga_exp.guolv.deg.gene1$logFC>0,])

cytoscape_down_gene=rownames(tcga_exp.guolv.deg.gene1[tcga_exp.guolv.deg.gene1$logFC<0,])

cytoscape_up<-data.frame(gene=cytoscape_up_gene,

type=rep('up',length(cytoscape_up_gene)))

cytoscape_down<-data.frame(gene=cytoscape_down_gene,

type=rep('down',length(cytoscape_down_gene)))

cytoscape<-rbind(cytoscape_up,cytoscape_down)

write.table(cytoscape,'result/PPI/lab.txt',sep='\t',quote = F)

#121个基因的热图

bk1=unique(c(seq(-1,1,length=100)))

cytoscape1=data.frame(type=cytoscape$type)

rownames(cytoscape1)=cytoscape$gene

sig_exp=tcga_exp.guolv[rownames(cytoscape1),group_subtype$sample]

anno_cols1<-list(clust=c('cluster1'='#E64B35','cluster2'='#4DBBD5'),

type=c('up'='red','down'='blue'))

pdf('result/PPI/121.gene.pheat.pdf',he=8,wi=15)

pheatmap(sig_exp, breaks = bk1,

scale = 'row',annotation_colors = anno_cols1,

annotation_col = col_anno,

annotation_row = cytoscape1,

cluster_cols = F, cluster_rows = T,

show_rownames = F, show_colnames = F,

color = colorRampPalette(c("navy", "white", "firebrick3"))(100))

dev.off()

#7个基因相关性分析

#CYP3A4、NR1I2、CYP2C9、TTR、APOC3、CYP1A2和AFP

gene_7=c('CYP3A4','NR1I2','CYP2C9','TTR','APOC3','CYP1A2','AFP')

gene_7.exp=t(sig_exp[gene_7,])

cor_matr = cor(gene_7.exp)

write.table(cor_matr, file="cor_matr.xls",row.names=F, col.names=T,quote=FALSE,sep="\t")

library(PerformanceAnalytics)

pdf('result/PPI/7_gene.cor.pdf',he=7,wi=7)

chart.Correlation(cor_matr,histogram = TRUE,pch=19)

dev.off()

#7个基因在两个亚型中分布情况

gene_7.exp.group<-merge(data.frame(sample=rownames(gene_7.exp),

gene_7.exp),

data.frame(sample=rownames(col_anno),

col_anno),by='sample')

head(gene_7.exp.group)

pdf('result/PPI/7_gene_subtype.pdf',he=8,wi=15)

groupViolin(data = log2(gene_7.exp.group[,2:8]+1),group = gene_7.exp.group$clust,group_col = c('cluster1'='#E64B35','cluster2'='#4DBBD5'),ylab = 'log2exp',xangle = 0)

dev.off()

#7个基因在临床特征中分布情况

gene_7.exp.cli<-merge(data.frame(sample=subtype.cli$sample,

T=subtype.cli$A3_T,

N=subtype.cli$A4_N,

stage=subtype.cli$A6_Stage,

grade=subtype.cli$A7_Grade),

data.frame(sample=rownames(gene_7.exp),

gene_7.exp),by='sample')

#T分期

gene_7.exp.cli.T<-gene_7.exp.cli[,-c(1,3,4,5)]

gene_7.exp.cli.T$T=gsub('a','',gene_7.exp.cli.T$T)

gene_7.exp.cli.T$T=gsub('b','',gene_7.exp.cli.T$T)

head(gene_7.exp.cli.T)

gene_7.exp.cli.T=gene_7.exp.cli.T[gene_7.exp.cli.T$T!='',]

unique(gene_7.exp.cli.T$T)

library(ggpubr)

library(data.table)

gene_7.exp.cli.T1<-melt(gene_7.exp.cli.T,id='T')

gene_7.exp.cli.T1$expression=log2(gene_7.exp.cli.T1$expression+1)

colnames(gene_7.exp.cli.T1)=c('Clust','gene','expression')

pdf('result/cli/T.pdf',wi=15,he=9)

Tplot=ggboxplot(gene_7.exp.cli.T1, x='gene', y='expression',

fill = "Clust", color = "black",

# palette = c("#00468B99","#42B54099"),

ylab="log2exp",xlab='',

add = "boxplot")+ rotate_x_text(angle = 0)+

stat_compare_means(aes(group=Clust),

symnum.args=list(cutpoints = c(0, 0.001, 0.01, 0.05, 1),

symbols = c("***", "**", "*", "ns")),label = "p.signif")

dev.off()

#N分期

gene_7.exp.cli.N<-gene_7.exp.cli[,-c(1,2,4,5)]

unique(gene_7.exp.cli.N$N)

library(ggpubr)

library(data.table)

gene_7.exp.cli.N1<-melt(gene_7.exp.cli.N,id='N')

colnames(gene_7.exp.cli.N1)=c('Clust','gene','expression')

gene_7.exp.cli.N1$expression=log2(gene_7.exp.cli.N1$expression+1)

pdf('result/cli/N.pdf',wi=15,he=9)

Nplot=ggboxplot(gene_7.exp.cli.N1, x='gene', y='expression',

fill = "Clust", color = "black",

# palette = c("#00468B99","#42B54099"),

ylab="log2exp",xlab='',

add = "boxplot")+ rotate_x_text(angle = 0)+

stat_compare_means(aes(group=Clust),

symnum.args=list(cutpoints = c(0, 0.001, 0.01, 0.05, 1),

symbols = c("***", "**", "*", "ns")),label = "p.signif")

dev.off()

#stage分期

gene_7.exp.cli.stage<-gene_7.exp.cli[,-c(1,2,3,5)]

unique(gene_7.exp.cli.stage$stage)

gene_7.exp.cli.stage$stage=gsub('A','',gene_7.exp.cli.stage$stage)

gene_7.exp.cli.stage$stage=gsub('B','',gene_7.exp.cli.stage$stage)

gene_7.exp.cli.stage$stage=gsub('C','',gene_7.exp.cli.stage$stage)

gene_7.exp.cli.stage=gene_7.exp.cli.stage[gene_7.exp.cli.stage$stage!='',]

library(ggpubr)

library(data.table)

gene_7.exp.cli.stage1<-melt(gene_7.exp.cli.stage,id='stage')

colnames(gene_7.exp.cli.stage1)=c('Clust','gene','expression')

gene_7.exp.cli.stage1$expression=log2(gene_7.exp.cli.stage1$expression+1)

pdf('result/cli/stage.pdf',wi=15,he=9)

stageplot=ggboxplot(gene_7.exp.cli.stage1, x='gene', y='expression',

fill = "Clust", color = "black",

# palette = c("#00468B99","#42B54099"),

ylab="log2exp",xlab='',

add = "boxplot")+ rotate_x_text(angle = 0)+

stat_compare_means(aes(group=Clust),

symnum.args=list(cutpoints = c(0, 0.001, 0.01, 0.05, 1),

symbols = c("***", "**", "*", "ns")),label = "p.signif")

dev.off()

#grade分期

gene_7.exp.cli.grade<-gene_7.exp.cli[,-c(1,2,3,4)]

unique(gene_7.exp.cli.grade$grade)

gene_7.exp.cli.grade=gene_7.exp.cli.grade[gene_7.exp.cli.grade$grade!='Not Available',]

library(ggpubr)

library(data.table)

gene_7.exp.cli.grade1<-melt(gene_7.exp.cli.grade,id='grade')

colnames(gene_7.exp.cli.grade1)=c('Clust','gene','expression')

gene_7.exp.cli.grade1$expression=log2(gene_7.exp.cli.grade1$expression+1)

pdf('result/cli/grade.pdf',wi=15,he=9)

gradeplot=ggboxplot(gene_7.exp.cli.grade1, x='gene', y='expression',

fill = "Clust", color = "black",

# palette = c("#00468B99","#42B54099"),

ylab="log2exp",xlab='',

add = "boxplot")+ rotate_x_text(angle = 0)+

stat_compare_means(aes(group=Clust),

symnum.args=list(cutpoints = c(0, 0.001, 0.01, 0.05, 1),

symbols = c("***", "**", "*", "ns")),label = "p.signif")

dev.off()

pdf('result/cli/all.pdf',he=15,wi=15)

ggpubr::ggarrange(Tplot,Nplot,stageplot,gradeplot

,ncol = 2,nrow = 2,

labels = toupper(letters)[1:4],align = "hv")

dev.off()

#生存分析

#过滤表达量为0的基因

tcga_exp.guolv<-tcga.exp[which(apply(tcga.exp,1,function(x){return(sum(x==0))})<ncol(tcga.exp)),]

dim(tcga_exp.guolv)

tcga_exp.guolv1=tcga_exp.guolv

rownames(tcga_exp.guolv1)=gsub('-','__',rownames(tcga_exp.guolv))

tcga_exp.guolv.time<-merge(data.frame(sample=paste0(gsub('-','\\.',

tcga_time$sample),'.01'),

OS=tcga_time$OS,

OS.time=tcga_time$OS.time),

data.frame(sample=colnames(tcga_exp.guolv1),

t(tcga_exp.guolv1)),by='sample')

#tcga_exp.guolv.time=na.omit(tcga_exp.guolv.time)

#rownames(tcga_exp.guolv.time)=tcga_exp.guolv.time$sample

tcga_exp.guolv.time2=tcga_exp.guolv.time[,c(2,3)]

tcga_exp.guolv.time1=t(tcga_exp.guolv.time[,-c(1,2,3)])

tcga_exp.guolv.time1=crbind2DataFrame(tcga_exp.guolv.time1)

tcga_exp.guolv.time1=na.omit(tcga_exp.guolv.time1)

all_gene_cox=cox_batch(log2(tcga_exp.guolv.time1+1),

tcga_exp.guolv.time2$OS.time,

tcga_exp.guolv.time2$OS)

all_gene_cox=na.omit(all_gene_cox)

all_gene_cox1=all_gene_cox

rownames(all_gene_cox1)=gsub('__','-',rownames(all_gene_cox1))

all_gene_cox1<-write.table(all_gene_cox1,'result/06gene_tongji/all_gene_cox.txt',sep='\t',quote = F)

#统计

table(all_gene_cox$p.value<0.05,all_gene_cox$HR>1)

#提取marker基因

rownames(all_gene_cox)=gsub('__','-',rownames(all_gene_cox))

com_gene1=intersect(com_gene,rownames(all_gene_cox))

mark_gene_cox=all_gene_cox[com_gene1,]

table(mark_gene_cox$p.value>=0.05)

write.table(mark_gene_cox,'result/06gene_tongji/mark_gene_cox.txt',sep='\t',quote=F)

#卡方检验

data1=t(matrix(c(8140,679,475,158,16179,1093),ncol=3))

chisq.test(data1)

deg_gene_cox=all_gene_cox[rownames(tcga_exp.guolv.deg.gene),]

#venn图

marker.gene=com_gene1

marker.gene.cox=rownames(mark_gene_cox[mark_gene_cox$p.value<0.05,])

marker.deg.gene=rownames(tcga_exp.guolv.deg.gene)

marker.deg.gene.cox=rownames(deg_gene_cox[deg_gene_cox$p.value<0.05,])

pdf('result/07deg_subtype/deg.marker.cox.venn.pdf',he=7,wi=7)

mg_venn_plot(list(marker.gene=marker.gene,

marker.gene.cox=marker.gene.cox,

marker.deg.gene=marker.deg.gene,

marker.deg.gene.cox=marker.deg.gene.cox))

dev.off()

#marker 差异基因，预后相关的226个基因

cox.clust.gene=intersect(marker.gene.cox,marker.deg.gene.cox)

tcga.surv=tcga.exp[cox.clust.gene,]

#，NMF聚类

library(NMF)

tcga_nmf <- nmf(log2(tcga.surv+1),2:10,nrun=100,seed=12345)

consensusmap(tcga_nmf,labCol=NA,labRow=NA,tracks=NA)

pdf('result/08.nmf/tcga_NMF_1.pdf',width = 8,height = 6)

plot(tcga_nmf)

dev.off()

tcga_nmf_2 <- nmf(log2(tcga.surv+1),

2,

nrun=100,

seed=12345)

pdf('result/08.nmf/tcga_NMF-2.pdf',width = 6,height = 6)

tcga_nmf_2_consensusmap <- consensusmap(tcga_nmf_2,

labCol=NA,

labRow=NA,

tracks=NA)

dev.off()

retrive_cluster_names <- function(myd,myd_consensusmap,hvalue){

sample_names<-rownames(myd)

myd_cut_list<-lapply(cut(myd_consensusmap$Colv,hvalue)$lower, function(l)rapply(l,function(i)i))

cluster_sample_names<-c()

tmp_cluster<-c()

c_index<- 1

for(i in myd_cut_list){

cluster_sample_names<-c(cluster_sample_names,as.character(sample_names[unlist(i)]))

tmp_cluster<-c(tmp_cluster,rep(paste("C",c_index,sep=""),length(unlist(i))))

c_index<-c_index+1

}

cluster_df<-data.frame("Sample"=cluster_sample_names,"Cluster"=tmp_cluster)

return(cluster_df);

}

tcga_nmf_2_cluster <- retrive_cluster_names(t(log2(tcga.surv+1)),

tcga_nmf_2_consensusmap,

0.6)

table(tcga_nmf_2_cluster$Cluster)

colnames(tcga_nmf_2_cluster)<-c("sample","cox_clust")

rownames(tcga_nmf_2_cluster) <- tcga_nmf_2_cluster[,1]

tcga_nmf_2_cluster$cox_clust<-gsub('C1','clust A',tcga_nmf_2_cluster$cox_clust)

tcga_nmf_2_cluster$cox_clust<-gsub('C2','clust B',tcga_nmf_2_cluster$cox_clust)

tcga_cli_nmf <- merge(tcga_subtype2,

tcga_nmf_2_cluster,

by = 'sample')

tcga_NMF_OS <- data.frame(tcga_cli_nmf$OS.time/365,

tcga_cli_nmf$OS,

tcga_cli_nmf$cox_clust)

tcga_NMF_OS <- na.omit(tcga_NMF_OS)

library(survival)

pdf('result/08.nmf/tcga_NMF_KM-3.pdf',width = 5,height = 5)

ggplotKMCox(tcga_NMF_OS,labs=c('clust A','clust B'))

dev.off()

#tcga_immnue.cell1,cox-clust两个亚型的免疫细胞组成

cox.clust.immnue.cell=merge(tcga_nmf_2_cluster,

data.frame(sample=rownames(tcga_immnue.cell1),

tcga_immnue.cell1),by='sample')

pdf('result/08.nmf/cox-clust.immnue.cell.pdf',he=15,wi=15)

groupViolin(data = cox.clust.immnue.cell[,-c(1,2)],

group = cox.clust.immnue.cell$cox_clust,

ylab = 'Immune Infiltration')

dev.off()

#保存表达谱数据

write.table(tcga.exp,'report/file/tcga.exp.LIHC.txt',sep='\t',quote = F)

write.table(subtype.cli,'report/file/cli_tcga.txt',sep='\t',quote=F)

library(data.table)

scRNA_exp<-fread('GSE149614_HCC.scRNAseq.S71915.normalized.txt',sep='\t',stringsAsFactors = F, na.strings = "", data.table = T)

save(scRNA_exp,file='scRNA_exp.RData')

library(dplyr)

library(Seurat)

library(stringr)

source('/pub1/data/mg_projects/projects/codes/mg_base.R')

#将正常cell和癌细胞分开

scRNA_exp=data.frame(scRNA_exp)

sample<-colnames(scRNA_exp)

rownames(scRNA_exp)=scRNA_exp$V1

sample_type<-str_split_fixed(sample,'_',n=2)[,1]

#肿瘤样本表达谱

#HCC10T.exp,HCC09T.exp,HCC08T.exp,HCC07T.exp,HCC06T.exp,HCC05T.exp,HCC04T.exp,HCC03T.exp,HCC02T.exp,HCC01T.exp

#正常样本表达谱

#HCC10N.exp,HCC09N.exp,HCC08N.exp,HCC07N.exp,HCC06N.exp,HCC05N.exp,HCC04N.exp,HCC03N.exp,

#淋巴结转移表达谱

#HCC10L.exp,

#HPV病毒感染

#HCC08P.exp,HCC07P.exp

sample.exp=function(data,sample_all,sample1){

name1=sample[which(sample_type == sample1)]

name1.exp=data[,name1]

return(name1.exp)

}

#肿瘤样本表达谱

HCC10T.exp=sample.exp(data = scRNA_exp,sample_all = sample,sample1 = 'HCC10T')

HCC09T.exp=sample.exp(data = scRNA_exp,sample_all = sample,sample1 = 'HCC09T')

HCC08T.exp=sample.exp(data = scRNA_exp,sample_all = sample,sample1 = 'HCC08T')

HCC07T.exp=sample.exp(data = scRNA_exp,sample_all = sample,sample1 = 'HCC07T')

HCC06T.exp=sample.exp(data = scRNA_exp,sample_all = sample,sample1 = 'HCC06T')

HCC05T.exp=sample.exp(data = scRNA_exp,sample_all = sample,sample1 = 'HCC05T')

HCC04T.exp=sample.exp(data = scRNA_exp,sample_all = sample,sample1 = 'HCC04T')

HCC03T.exp=sample.exp(data = scRNA_exp,sample_all = sample,sample1 = 'HCC03T')

HCC02T.exp=sample.exp(data = scRNA_exp,sample_all = sample,sample1 = 'HCC02T')

HCC01T.exp=sample.exp(data = scRNA_exp,sample_all = sample,sample1 = 'HCC01T')

#正常样本的表达谱

HCC10N.exp=sample.exp(data = scRNA_exp,sample_all = sample,sample1 = 'HCC10N')

HCC09N.exp=sample.exp(data = scRNA_exp,sample_all = sample,sample1 = 'HCC09N')

HCC08N.exp=sample.exp(data = scRNA_exp,sample_all = sample,sample1 = 'HCC08N')

HCC07N.exp=sample.exp(data = scRNA_exp,sample_all = sample,sample1 = 'HCC07N')

HCC06N.exp=sample.exp(data = scRNA_exp,sample_all = sample,sample1 = 'HCC06N')

HCC05N.exp=sample.exp(data = scRNA_exp,sample_all = sample,sample1 = 'HCC05N')

HCC04N.exp=sample.exp(data = scRNA_exp,sample_all = sample,sample1 = 'HCC04N')

HCC03N.exp=sample.exp(data = scRNA_exp,sample_all = sample,sample1 = 'HCC03N')

#淋巴结转移表达谱

HCC10L.exp=sample.exp(data = scRNA_exp,sample_all = sample,sample1 = 'HCC10L')

#HPV病毒感染

HCC08P.exp=sample.exp(data = scRNA_exp,sample_all = sample,sample1 = 'HCC08P')

HCC07P.exp=sample.exp(data = scRNA_exp,sample_all = sample,sample1 = 'HCC07P')

#对肿瘤和正常细胞进行处理

#scRNA_exp_all<-cbind(HCC10T.exp,HCC09T.exp,HCC08T.exp,HCC07T.exp,HCC06T.exp,HCC05T.exp,HCC04T.exp,

#HCC03T.exp,HCC02T.exp,HCC01T.exp,HCC10N.exp,HCC09N.exp,HCC08N.exp,HCC07N.exp,HCC06N.exp,HCC05N.exp,HCC04N.exp,HCC03N.exp)

scRNA_exp_all<-cbind(HCC10T.exp,HCC09T.exp,HCC08T.exp,HCC07T.exp,HCC06T.exp,HCC05T.exp,HCC04T.exp,

HCC03T.exp,HCC02T.exp,HCC01T.exp)

pbmcT <- CreateSeuratObject(counts = scRNA_exp_all, project = "seurat", min.cells = 100, min.features = 2000)

#pbmc@meta.data

pbmcT

#An object of class Seurat

#17093 features across 16880 samples within 1 assay

#Active assay: RNA (17093 features)

#认为以MT开头的为线粒体

pbmcT[["percent.mt"]] <- PercentageFeatureSet(pbmcT, pattern = "^MT-")

#head(pbmcT@meta.data)

#可视化QC指标，并用它们来过滤细胞

pdf('scRNA/1.pdf',wi=15,he=9)

VlnPlot(pbmcT, features = c("nFeature_RNA", "nCount_RNA", "percent.mt"),ncol = 3)

dev.off()

#另外可以用一个散点图（FeatureScatter）来绘制两组feature信息的相关性，最后组合在一起（CombinePlots）

plot1 <- FeatureScatter(pbmcT, feature1 = "nCount_RNA", feature2 = "percent.mt")

plot2 <- FeatureScatter(pbmcT, feature1 = "nCount_RNA", feature2 = "nFeature_RNA")

pdf('scRNA/2.pdf',wi=9,he=9)

CombinePlots(plots = list(plot1, plot2))

dev.off()

#过滤

pbmcT1 <- subset(pbmcT, subset = nFeature_RNA < 8000 & nCount_RNA>2000 & percent.mt < 2.5)

pdf('scRNA/guolv1.pdf',wi=15,he=9)

VlnPlot(pbmcT1, features = c("nFeature_RNA", "nCount_RNA", "percent.mt"),ncol = 3)

dev.off()

pbmcT1

#An object of class Seurat

#17396 features across 19656 samples within 1 assay

#Active assay: RNA (17396 features)

保存文件

write.table(pbmcT1[["RNA"]]@data,'scRNA/sample.exp.txt',sep='\t',quote=F)

plot1 <- FeatureScatter(pbmcT1, feature1 = "nCount_RNA", feature2 = "percent.mt")

plot2 <- FeatureScatter(pbmcT1, feature1 = "nCount_RNA", feature2 = "nFeature_RNA")

pdf('scRNA/guolv2.pdf',wi=9,he=9)

CombinePlots(plots = list(plot1, plot2))

dev.off()

#数据归一化处理,结果存储在pbmc2[["RNA"]]@data

#如果是TPM的矩阵就不能使用NormalizeData，直接取log降维即可

#pbmcT1 <- NormalizeData(pbmcT1)

#pbmcT1<-log2(pbmcT1[["RNA"]]@data+1)

#鉴定差异基因HVGs(高变异基因)

pbmcT2 <- FindVariableFeatures(pbmcT1, selection.method = "vst", nfeatures = 2000)

pbmcT2=as.matrix(pbmcT2)

top10 <- head(VariableFeatures(pbmcT2), 10)

# 分别绘制带基因名和不带基因名的

plot1 <- VariableFeaturePlot(pbmcT2)

plot2 <- LabelPoints(plot = plot1, points = top10, repel = TRUE)

pdf('scRNA/4.pdf',wi=15,he=9)

CombinePlots(plots = list(plot1, plot2))

dev.off()

#数据的标准化

# 先使用全部基因

all.genes <- rownames(pbmcT2)

length(all.genes)

#全部全部基因进行标准化，使用的是z-score的标准化的方法

pbmcT3 <- ScaleData(pbmcT2, features = all.genes)

#PCA降维，结果保存在reductions

pbmcT4 <- RunPCA(pbmcT3, features = VariableFeatures(object = pbmcT3))

print(pbmcT4[["pca"]], dims = 1:20, nfeatures = 5)

#PCA可视化

pdf('scRNA/5.pdf',wi=9,he=15)

VizDimLoadings(pbmcT4, dims = 1:5, reduction = "pca")

dev.off()

#用DimPlot进行降维后的可视化,默认用的是前两个

pdf('scRNA/6.pdf',wi=7,he=7)

DimPlot(pbmcT4, reduction = "pca")

dev.off()

#探索异质性来源

pdf('scRNA/7.pdf',wi=15,he=15)

DimHeatmap(pbmcT4, dims = 1:15, cells = 500, balanced = TRUE)

dev.off()

#降维之后查看选取多少个PC可以代表整个数据集,它是根据每个主成分对总体变异水平的贡献百分比排序得到的图，我们主要关注”肘部“的PC，它是一个转折点（也即是这里的PC10-11），说明取前11个主成分可以比较好地代表总体变化

pdf('scRNA/8.pdf',wi=15,he=15)

ElbowPlot(pbmcT4,ndims = 50)

dev.off()

#取前11个主成分做细胞聚类

pbmcT5 <- FindNeighbors(pbmcT4, dims = 1:11)

pbmcT6 <- FindClusters(pbmcT5, resolution = 0.3,random.seed=123456,algorithm=1)

head(Idents(pbmcT6), 5)

#cluster0.markers <- FindMarkers(pbmcT6, ident.1 = 0, min.pct = 0.25)

#cluster1.markers <- FindMarkers(pbmcT6, ident.1 = 1, min.pct = 0.25)

#cluster2.markers <- FindMarkers(pbmcT6, ident.1 = 2, min.pct = 0.25)

#cluster3.markers <- FindMarkers(pbmcT6, ident.1 = 3, min.pct = 0.25)

#cluster4.markers <- FindMarkers(pbmcT6, ident.1 = 4, min.pct = 0.25)

#cluster5.markers <- FindMarkers(pbmcT6, ident.1 = 5, min.pct = 0.25)

#write.table(cluster0.markers,'cluster0.markers.T.txt',sep='\t',quote=F)

#write.table(cluster1.markers,'cluster1.markers.T.txt',sep='\t',quote=F)

#write.table(cluster2.markers,'cluster2.markers.T.txt',sep='\t',quote=F)

#write.table(cluster3.markers,'cluster3.markers.T.txt',sep='\t',quote=F)

#write.table(cluster4.markers,'cluster4.markers.T.txt',sep='\t',quote=F)

#write.table(cluster5.markers,'cluster5.markers.T.txt',sep='\t',quote=F)

pbmcT7 <- RunUMAP(pbmcT6, dims = 1:11)

pdf('scRNA/9.pdf',wi=7,he=7)

DimPlot(pbmcT7, reduction = "umap",label = TRUE) # 还可以设置label = TRUE让数字显示在每个cluster上

dev.off()

cluster.all.markers <- FindAllMarkers(pbmcT7,min.pct = 0.5,logfc.threshold = 0.25)

markers<-subset(cluster.all.markers, cluster.all.markers$avg_logFC > 1 | cluster.all.markers$avg_logFC=='Inf')

write.table(markers,'scRNA/cluster.all.markers.txt',sep='\t',quote=F,row.names=FALSE)

clust_sample<-pbmcT7@meta.data

write.table(pbmcT7@meta.data,'scRNA/clust_sample_T.txt',sep='\t',quote=F)

#HCL细胞类型预测####

sampe2=as.character(rownames(pbmcT7@meta.data))

scRNA_cell_type=scRNA_exp_all[markers$gene,sampe2]

write.table(scRNA_cell_type,'scRNA/cell.type/scRNA_cell_type.txt',sep='\t',quote=F)

library(scHCL)

hcl_result <- scHCL(scdata = scRNA_cell_type, numbers_plot = 3)

data_test <- data.frame(samples = names(hcl_result$scHCL), cell = hcl_result$scHCL)

write.table(data_test,'hcl_cell_types.txt',sep='\t',quote=F)

hcl_cell_all=data.frame(hcl_result$cors_matrix)

scHCL

#hcl_cell_types=c()

#for (i in 1:dim(hcl_cell_all)[2]){

#cell_name=rownames(hcl_cell_all[hcl_cell_all[,i]==max(hcl_cell_all[,i]),])

#hcl_cell_type=data.frame(sample=colnames(hcl_cell_all)[i],cell=cell_name)

#hcl_cell_types=rbind(hcl_cell_types,hcl_cell_type)

#}

#write.table(hcl_cell_types,'hcl_cell_types.txt',sep='\t',quote=F)

write.table(hcl_cell_all,'hcl_cell_all.txt',sep='\t',quote=F)

#save(hcl_cell_types,file='hcl_cell_types.RData')

save(hcl_cell_all,file='hcl_cell_all.RData')

#细胞轨迹分析####

library(monocle)

library(BiocGenerics)

# ---------首先构建基因的注释信息(feature_data)

gene_ann <- data.frame(gene_short_name = row.names(pbmcT7[["RNA"]]@data), row.names = row.names(pbmcT7[["RNA"]]@data))

fd <- new("AnnotatedDataFrame", data=gene_ann)

# 然后构建样本的注释信息(sample_data)

sample1=colnames(pbmcT7)

sample1_type=str_split_fixed(sample1,'_',n=2)[,1]

sample_ann<-data.frame(sample=sample1,patient=sample1_type)

rownames(sample_ann)=sample_ann$sample

pd <- new("AnnotatedDataFrame",data=sample_ann)

# 开始构建对象

sc_cds <- newCellDataSet(as.matrix(pbmcT7[["RNA"]]@data), phenoData = pd,featureData =fd,expressionFamily = negbinomial.size(),lowerDetectionLimit=1)

sc_cds

cds=sc_cds

cds <- detectGenes(cds, min_expr = 0.1)

print(head(cds@featureData@data))

expressed_genes <- row.names(subset(cds@featureData@data,num_cells_expressed >= 5))

length(expressed_genes)

# 17093

# 这里需要去掉ERCC基因

# 去掉ERCC基因

is.ercc <- grepl("ERCC",expressed_genes)

length(expressed_genes[!is.ercc])

# 17084(看到去掉了9个ERCC)

cds <- cds[expressed_genes[!is.ercc],]

cds

# 过滤基因后是 17084 features, 16601 samples

#然后是对细胞的过滤

# 如果不支持使用pData()函数，可以使用cds@phenoData@data来获得各种细胞注释信息

cell_anno <- cds@phenoData@data

head(cell_anno)

# 这里简单过滤细胞

valid_cells <- row.names(cell_anno[cell_anno$num_genes_expressed>1000,] )

cds <- cds[,valid_cells]

cds

# 最后剩下： 17084 features, 16601 samples

### 必要的归一化

library(dplyr)

colnames(phenoData(cds)@data)

cds <- estimateSizeFactors(cds)

cds <- estimateDispersions(cds)

#降维聚类

disp_table <- dispersionTable(cds)

unsup_clustering_genes <- subset(disp_table, mean_expression >= 0.05)

cds <- setOrderingFilter(cds, unsup_clustering_genes$gene_id)

cds

# 图中黑色的点就是被标记出来一会要进行聚类的基因

pdf('monocle/1.pdf',wi=7,he=7)

cds <- reduceDimension(cds, max_components = 2,reduction_method = 'tSNE', verbose = T)

# 进行聚类num_clusters可以修改的

cds1 <- clusterCells(cds, num_clusters = 21)

#Distance cutoff calculated to 1.346129

# color使用的这些数据就在：cds$Cluster

pdf('monocle/1.pdf',wi=7,he=7)

plot_cell_clusters(cds1, 1, 2, color = "Cluster")

dev.off()

cds2 <- reduceDimension(cds1, max_components = 2,reduction_method = 'tSNE',residualModelFormulaStr = "~num_genes_expressed",verbose = T)

cds2 <- clusterCells(cds2, num_clusters = 21)

pdf('monocle/2.pdf',wi=7,he=7)

plot_cell_clusters(cds2, 1, 2, color = "Cluster")

dev.off()

#选合适基因 => setOrderingFilter()#####

ordering_genes <- unique(markers$gene)

cds3 <- setOrderingFilter(cds2, ordering_genes)

pdf('monocle/3.pdf',wi=7,he=7)

plot_ordering_genes(cds3)

dev.off()

#降维 => reduceDimension(

cds3 <- reduceDimension(cds3, max_components = 2,method = 'DDRTree')

#细胞排序 => orderCells()

cds3 <- orderCells(cds3)

#可视化

pdf('monocle/4.pdf',wi=7,he=7)

plot_cell_trajectory(cds3, color_by = "Cluster")

dev.off()

write.table(cds3@phenoData@data,'monocle/monocle.clust.txt',sep='\t',quote=F)

clust.sample=data.frame(sample=cds3@phenoData@data$sample,cluster=cds3@phenoData@data$Cluster)

rownames(clust.sample)=clust.sample$sample

#对每一个clust进行相关性分析

clust1.sample=rownames(clust.sample[clust.sample$cluster=='1',])

clust2.sample=rownames(clust.sample[clust.sample$cluster=='2',])

clust3.sample=rownames(clust.sample[clust.sample$cluster=='3',])

clust4.sample=rownames(clust.sample[clust.sample$cluster=='4',])

clust5.sample=rownames(clust.sample[clust.sample$cluster=='5',])

clust6.sample=rownames(clust.sample[clust.sample$cluster=='6',])

clust7.sample=rownames(clust.sample[clust.sample$cluster=='7',])

clust8.sample=rownames(clust.sample[clust.sample$cluster=='8',])

clust9.sample=rownames(clust.sample[clust.sample$cluster=='9',])

clust10.sample=rownames(clust.sample[clust.sample$cluster=='10',])

clust11.sample=rownames(clust.sample[clust.sample$cluster=='11',])

clust12.sample=rownames(clust.sample[clust.sample$cluster=='12',])

clust13.sample=rownames(clust.sample[clust.sample$cluster=='13',])

clust14.sample=rownames(clust.sample[clust.sample$cluster=='14',])

clust15.sample=rownames(clust.sample[clust.sample$cluster=='15',])

clust16.sample=rownames(clust.sample[clust.sample$cluster=='16',])

clust17.sample=rownames(clust.sample[clust.sample$cluster=='17',])

clust18.sample=rownames(clust.sample[clust.sample$cluster=='18',])

clust19.sample=rownames(clust.sample[clust.sample$cluster=='19',])

clust20.sample=rownames(clust.sample[clust.sample$cluster=='20',])

#cluster1 相关性分析

clust1.exp=t(scRNA_exp_all[ordering_genes,clust1.sample])

clust1.exp.cox=cor(clust1.exp)

library(tidyr)

clust1.exp.cox1=data.frame(gene1=rownames(clust1.exp.cox),clust1.exp.cox)

clust1.exp.cox2 <- gather(clust1.exp.cox1, gene2, cor, -gene1)

#去重，去掉相关性为1的关系对

clust1.exp.cox2=clust1.exp.cox2[clust1.exp.cox2$cor!=1,]
