# Supplementary material for: Identification and Characterization of Genes Related to the Prognosis of Hepatocellular Carcinoma Based on Single-Cell Sequencing
Source: Pathol Oncol Res. 2022 Aug 25;28:1610199. doi: 10.3389/pore.2022.1610199 (PMC9454301; doi:10.3389/pore.2022.1610199)
Supplement: Supplementary file 11 [file Table2.DOCX]

**Table 2. The cell comparison of 20 sub-clusters from single-cell sequencing.**

|  | **Cells** | **Number** | **Proportion** |
| --- | --- | --- | --- |
| **Clust1** | Hepatocyte.Adult.Liver4. | 1365 | 0.966029724 |
|  | Hepatocyte_FGB.high.Adult.Liver1. | 31 | 0.021939137 |
|  | Hepatocyte_HP.high.Adult.Liver2. | 17 | 0.012031139 |
| **Clust2** | Hepatocyte.Adult.Liver4. | 435 | 0.524728589 |
|  | Hepatocyte_HP.high.Adult.Liver2. | 123 | 0.148371532 |
|  | Epithelial.cell_TM4SF4.high.Adult.Liver2. | 87 | 0.104945718 |
|  | Hepatocyte_FGB.high.Adult.Liver1. | 64 | 0.077201448 |
|  | Hepatocyte_APOA2.high.Adult.Liver2. | 47 | 0.056694813 |
|  | M3.Placenta_VentoTormo. | 41 | 0.049457177 |
|  | Epithelial.cell.Adult.Liver4. | 10 | 0.012062726 |
|  | PV1.Placenta_VentoTormo. | 8 | 0.009650181 |
|  | Epi.Placenta_VentoTormo. | 6 | 0.007237636 |
|  | Adult.hepatocyte.Liver_Camp. | 5 | 0.006031363 |
|  | Endo..m..Placenta_VentoTormo. | 1 | 0.001206273 |
|  | Epithelial.cell.Adult.Gall.Bladder1. | 1 | 0.001206273 |
|  | Smooth.muscle.cell.Adult.Adrenal.Gland2. | 1 | 0.001206273 |
| **Clust3** | Hepatocyte.Adult.Liver4. | 1501 | 0.984262295 |
|  | Hepatocyte_HP.high.Adult.Liver2. | 24 | 0.015737705 |
| **Clust4** | Hepatocyte.Adult.Liver4. | 913 | 0.98172043 |
|  | Hepatocyte_HP.high.Adult.Liver2. | 14 | 0.015053763 |
|  | Hepatocyte_FGB.high.Adult.Liver1. | 3 | 0.003225806 |
| **Clust5** | Hepatocyte.Adult.Liver4. | 828 | 0.975265018 |
|  | Hepatocyte_HP.high.Adult.Liver2. | 21 | 0.024734982 |
| **Clust6** | Hepatocyte.Adult.Liver4. | 1799 | 0.868662482 |
|  | Hepatocyte_FGB.high.Adult.Liver1. | 263 | 0.126991791 |
|  | Hepatocyte_HP.high.Adult.Liver2. | 8 | 0.003862868 |
|  | M3.Placenta_VentoTormo. | 1 | 0.000482859 |
| **Clust7** | Endo..m..Placenta_VentoTormo. | 608 | 0.446074835 |
|  | Endothelial.cell.Testis_Guo. | 270 | 0.198092443 |
|  | Vascular.endothelial.cell.Adult.Liver2. | 175 | 0.12839325 |
|  | Hepatocyte.Adult.Liver4. | 152 | 0.111518709 |
|  | Sinusoidal.endothelial.cell.Adult.Liver2. | 32 | 0.023477623 |
|  | Endothelial.cell_CCL2.high.Adult.Adrenal.Gland3. | 28 | 0.02054292 |
|  | Hepatocyte_APOA2.high.Adult.Liver2. | 23 | 0.016874541 |
|  | Endothelial.cell_IGFBP5.high.Neonatal.Adrenal.Gland1. | 22 | 0.016140866 |
|  | Hepatocyte_FGB.high.Adult.Liver1. | 12 | 0.008804109 |
|  | Endothelial.cell_ACKR1.high.Adult.Heart2. | 11 | 0.008070433 |
|  | Hepatocyte_HP.high.Adult.Liver2. | 9 | 0.006603081 |
|  | PV1.Placenta_VentoTormo. | 5 | 0.003668379 |
|  | Endo.L.Placenta_VentoTormo. | 3 | 0.002201027 |
|  | Smooth.muscle.cell.Adult.Heart2. | 2 | 0.001467351 |
|  | Vascular.endothelial.cell.Adult.Liver4. | 2 | 0.001467351 |
|  | Endothelial.cell_A2M.high.Fetal.Pancreas1. | 1 | 0.000733676 |
|  | Endothelial.cell_IGFBP5.high.Adult.Kidney4. | 1 | 0.000733676 |
|  | Endothelial.cell_SPARCL1.high.Adult.Lung3. | 1 | 0.000733676 |
|  | Neutrophil.Adult.Heart2. | 1 | 0.000733676 |
|  | Sinusoidal.endothelial.cell.Adult.Liver1. | 1 | 0.000733676 |
|  | Smooth.muscle.cell.Adult.Heart1. | 1 | 0.000733676 |
|  | Smooth.muscle.cell_CYCS.high.Adult.Heart2. | 1 | 0.000733676 |
|  | T.cell.Adult.Heart2. | 1 | 0.000733676 |
|  | Ventricle.cardiomyocyte.Adult.Heart1. | 1 | 0.000733676 |
| **Clust8** | Hepatocyte_FGB.high.Adult.Liver1. | 366 | 0.508333333 |
|  | Hepatocyte.Adult.Liver4. | 346 | 0.480555556 |
|  | Fetal.hepatocyte.Liver_Camp. | 2 | 0.002777778 |
|  | Epithelial.cell.Adult.Gall.Bladder1. | 1 | 0.001388889 |
|  | Epithelial.cell.Adult.Liver4. | 1 | 0.001388889 |
|  | Hepatocyte_APOA2.high.Adult.Liver2. | 1 | 0.001388889 |
|  | Hepatocyte_HP.high.Adult.Liver2. | 1 | 0.001388889 |
|  | Luminal.cell_CD74.high.Breast.Epithelium_Nguyen. | 1 | 0.001388889 |
|  | Mucous.epithelial.cell_TFF1.high.Adult.Gall.Bladder1. | 1 | 0.001388889 |
| **Clust9** | Kupffer.Cell.Adult.Liver4. | 158 | 0.323770492 |
|  | dM1.Placenta_VentoTormo. | 151 | 0.30942623 |
|  | M3.Placenta_VentoTormo. | 94 | 0.192622951 |
|  | dM2.Placenta_VentoTormo. | 63 | 0.129098361 |
|  | DC1.Placenta_VentoTormo. | 12 | 0.024590164 |
|  | Kupffer.cell_C1QB.high.Adult.Liver2. | 6 | 0.012295082 |
|  | Myeloid.cell.Adult.Liver4. | 2 | 0.004098361 |
|  | Dendritic.cell.Adult.Liver4. | 1 | 0.00204918 |
|  | Neutrophil.Adult.Heart2. | 1 | 0.00204918 |
| **Clust10** | Hepatocyte.Adult.Liver4. | 433 | 0.638643068 |
|  | Hepatocyte_FGB.high.Adult.Liver1. | 242 | 0.356932153 |
|  | Hepatocyte_HP.high.Adult.Liver2. | 3 | 0.004424779 |
| **Clust11** | Kupffer.Cell.Adult.Liver4. | 163 | 0.396593674 |
|  | M3.Placenta_VentoTormo. | 159 | 0.386861314 |
|  | Hepatocyte_HP.high.Adult.Liver2. | 82 | 0.199513382 |
|  | Hepatocyte.Adult.Liver4. | 6 | 0.01459854 |
|  | Hepatocyte_APOA2.high.Adult.Liver2. | 1 | 0.00243309 |
| **Clust12** | Hepatocyte.Adult.Liver4. | 281 | 0.552062868 |
|  | Hepatocyte_FGB.high.Adult.Liver1. | 54 | 0.106090373 |
|  | Epi.Placenta_VentoTormo. | 47 | 0.092337917 |
|  | M3.Placenta_VentoTormo. | 35 | 0.068762279 |
|  | Epithelial.cell_TM4SF4.high.Adult.Liver2. | 32 | 0.062868369 |
|  | Epithelial.cell.Adult.Liver4. | 21 | 0.041257367 |
|  | Hepatocyte_HP.high.Adult.Liver2. | 14 | 0.027504912 |
|  | Hepatocyte_APOA2.high.Adult.Liver2. | 9 | 0.017681729 |
|  | Adult.hepatocyte.Liver_Camp. | 6 | 0.011787819 |
|  | Unknown.Adult.Kidney3. | 2 | 0.003929273 |
|  | Basal.cell_KRT17.high.Breast.Epithelium_Nguyen. | 1 | 0.001964637 |
|  | Contaminated.cell.Breast.Epithelium_Nguyen. | 1 | 0.001964637 |
|  | dM1.Placenta_VentoTormo. | 1 | 0.001964637 |
|  | dS5.Placenta_VentoTormo. | 1 | 0.001964637 |
|  | Luminal.cell_AGR2.high.Breast.Epithelium_Nguyen. | 1 | 0.001964637 |
|  | Proximal.tubule.cell_MT1G.high.Adult.Kidney3. | 1 | 0.001964637 |
|  | PV1.Placenta_VentoTormo. | 1 | 0.001964637 |
|  | T.cell.Adult.Kidney2. | 1 | 0.001964637 |
| **Clust13** | T.cells3.Placenta_VentoTormo. | 95 | 0.25198939 |
|  | Hepatocyte_APOA2.high.Adult.Liver2. | 83 | 0.220159151 |
|  | T.cells4.Placenta_VentoTormo. | 82 | 0.217506631 |
|  | Hepatocyte.Adult.Liver4. | 68 | 0.180371353 |
|  | Activated.T.cell.Adult.Liver4. | 21 | 0.055702918 |
|  | Hepatocyte_HP.high.Adult.Liver2. | 13 | 0.034482759 |
|  | T.cells1.Placenta_VentoTormo. | 4 | 0.01061008 |
|  | Hepatocyte_FGB.high.Adult.Liver1. | 3 | 0.00795756 |
|  | dM1.Placenta_VentoTormo. | 2 | 0.00530504 |
|  | dNK2.Placenta_VentoTormo. | 2 | 0.00530504 |
|  | Activated.T.cell.Adult.Liver2. | 1 | 0.00265252 |
|  | Smooth.muscle.cell.Adult.Liver2. | 1 | 0.00265252 |
|  | T.cell.Adult.Lung3. | 1 | 0.00265252 |
|  | Vascular.endothelial.cell.Adult.Liver2. | 1 | 0.00265252 |
| **Clust14** | Hepatocyte_FGB.high.Adult.Liver1. | 223 | 0.518604651 |
|  | Hepatocyte.Adult.Liver4. | 201 | 0.46744186 |
|  | Hepatocyte_HP.high.Adult.Liver2. | 4 | 0.009302326 |
|  | Mucous.epithelial.cell_TFF1.high.Adult.Gall.Bladder1. | 2 | 0.004651163 |
| **Clust15** | Kupffer.Cell.Adult.Liver4. | 524 | 0.54697286 |
|  | M3.Placenta_VentoTormo. | 104 | 0.108559499 |
|  | DC1.Placenta_VentoTormo. | 103 | 0.107515658 |
|  | dM1.Placenta_VentoTormo. | 53 | 0.055323591 |
|  | Hepatocyte_APOA2.high.Adult.Liver2. | 45 | 0.04697286 |
|  | Dendritic.cell.Adult.Liver4. | 29 | 0.030271399 |
|  | Hepatocyte_HP.high.Adult.Liver2. | 26 | 0.027139875 |
|  | dM2.Placenta_VentoTormo. | 17 | 0.017745303 |
|  | Kupffer.cell_C1QB.high.Adult.Liver2. | 15 | 0.01565762 |
|  | Conventional.dendritic.cell.Adult.Liver4. | 11 | 0.011482255 |
|  | Dendritic.cell.Adult.Liver2. | 8 | 0.008350731 |
|  | Macrophage.Testis_Guo. | 7 | 0.007306889 |
|  | Hepatocyte.Adult.Liver4. | 5 | 0.005219207 |
|  | Myeloid.cell.Adult.Liver4. | 4 | 0.004175365 |
|  | Macrophage.Adult.Ileum2. | 3 | 0.003131524 |
|  | Conventional.dendritic.cell_FECER1A.high.Adult.Liver2. | 1 | 0.001043841 |
|  | Smooth.muscle.cell.Adult.Adrenal.Gland2. | 1 | 0.001043841 |
|  | Vascular.endothelial.cell.Adult.Liver2. | 1 | 0.001043841 |
|  | Vascular.endothelial.cell.Adult.Liver4. | 1 | 0.001043841 |
| **Clust16** | M3.Placenta_VentoTormo. | 766 | 0.619240097 |
|  | Kupffer.Cell.Adult.Liver4. | 285 | 0.23039612 |
|  | dM1.Placenta_VentoTormo. | 89 | 0.071948262 |
|  | Hepatocyte_HP.high.Adult.Liver2. | 38 | 0.030719483 |
|  | dM2.Placenta_VentoTormo. | 25 | 0.020210186 |
|  | DC1.Placenta_VentoTormo. | 15 | 0.012126112 |
|  | Dendritic.cell..Placenta_Tsang. | 5 | 0.004042037 |
|  | Kupffer.cell_C1QB.high.Adult.Liver2. | 4 | 0.00323363 |
|  | Hepatocyte.Adult.Liver4. | 3 | 0.002425222 |
|  | Myeloid.cell.Adult.Liver4. | 2 | 0.001616815 |
|  | Conventional.dendritic.cell_FECER1A.high.Adult.Liver2. | 1 | 0.000808407 |
|  | Dendritic.cell.Adult.Liver4. | 1 | 0.000808407 |
|  | Macrophage.Adult.Adrenal.Gland3..1 | 1 | 0.000808407 |
|  | Macrophage_CCL3L3.high.Adult.Heart1. | 1 | 0.000808407 |
|  | Vascular.endothelial.cell.Adult.Liver2. | 1 | 0.000808407 |
| **Clust17** | Hepatocyte.Adult.Liver4. | 534 | 0.988888889 |
|  | Hepatocyte_HP.high.Adult.Liver2. | 6 | 0.011111111 |
| **Clust18** | PV1.Placenta_VentoTormo. | 733 | 0.91625 |
|  | Hepatocyte.Adult.Liver4. | 12 | 0.015 |
|  | Hepatocyte_APOA2.high.Adult.Liver2. | 7 | 0.00875 |
|  | Neutrophil.Adult.Heart2. | 6 | 0.0075 |
|  | Macrophage_CCL3L3.high.Adult.Heart1. | 5 | 0.00625 |
|  | Hepatocyte_HP.high.Adult.Liver2. | 4 | 0.005 |
|  | Smooth.muscle.cell.Adult.Heart1. | 4 | 0.005 |
|  | Vascular.smooth.cell.Placenta_Tsang. | 4 | 0.005 |
|  | Dendritic.cell..Placenta_Tsang. | 3 | 0.00375 |
|  | M3.Placenta_VentoTormo. | 3 | 0.00375 |
|  | Smooth.muscle.cell.Adult.Heart2. | 3 | 0.00375 |
|  | dS5.Placenta_VentoTormo. | 2 | 0.0025 |
|  | Endo..m..Placenta_VentoTormo. | 2 | 0.0025 |
|  | Kupffer.Cell.Adult.Liver4. | 2 | 0.0025 |
|  | M2.Macrophage.Adult.Heart2. | 2 | 0.0025 |
|  | Dendritic.cell.Placenta1. | 1 | 0.00125 |
|  | Macrophage.Adult.Adrenal.Gland3..1 | 1 | 0.00125 |
|  | Macrophage.Adult.Heart2. | 1 | 0.00125 |
|  | Smooth.muscle.cell.Adult.Liver2. | 1 | 0.00125 |
|  | Smooth.muscle.cell.Adult.Lung3. | 1 | 0.00125 |
|  | Smooth.muscle.cell.Adult.Thyroid2. | 1 | 0.00125 |
|  | T.cell.Adult.Heart2. | 1 | 0.00125 |
|  | Ventricle.cardiomyocyte.Adult.Heart1. | 1 | 0.00125 |
| **Clust19** | Hepatocyte_APOA2.high.Adult.Liver2. | 148 | 0.548148148 |
|  | Hepatocyte.Adult.Liver4. | 86 | 0.318518519 |
|  | Hepatocyte_HP.high.Adult.Liver2. | 10 | 0.037037037 |
|  | Activated.T.cell.Adult.Liver2. | 4 | 0.014814815 |
|  | Activated.T.cell.Adult.Liver4. | 3 | 0.011111111 |
|  | B.cell..Plasmocyte..Adult.Adrenal.Gland3. | 3 | 0.011111111 |
|  | B.cell..Plasmocyte._IGHG.high.Adult.Liver2. | 3 | 0.011111111 |
|  | Hepatocyte_FGB.high.Adult.Liver1. | 3 | 0.011111111 |
|  | DC1.Placenta_VentoTormo. | 2 | 0.007407407 |
|  | Dendritic.cell.Adult.Liver4. | 2 | 0.007407407 |
|  | M3.Placenta_VentoTormo. | 2 | 0.007407407 |
|  | T.cells4.Placenta_VentoTormo. | 2 | 0.007407407 |
|  | dM1.Placenta_VentoTormo. | 1 | 0.003703704 |
|  | Epithelial.cell_TM4SF4.high.Adult.Liver2. | 1 | 0.003703704 |
| **Clust20** | Hepatocyte.Adult.Liver4. | 194 | 0.955665025 |
|  | Hepatocyte_HP.high.Adult.Liver2. | 9 | 0.044334975 |
